# Identification of Oxygenated Fatty Acid as a Side Chain of Lipo-Alkaloids in *Aconitum carmichaelii* by UHPLC-Q-TOF-MS and a Database

**DOI:** 10.3390/molecules21040437

**Published:** 2016-03-31

**Authors:** Ying Liang, Jian-Lin Wu, Elaine Lai-Han Leung, Hua Zhou, Zhongqiu Liu, Guanyu Yan, Ying Liu, Liang Liu, Na Li

**Affiliations:** 1 State Key Laboratory of Quality Research in Chinese Medicine, Macau Institute for Applied Research in Medicine and Health, Macau University of Science and Technology, Avenida Wai Long, Taipa, Macao, China; 1309853gct20002@student.must.edu.mo (Y.L.); jlwu@must.edu.mo (J.-L.W.); lhleung@must.edu.mo (E.L.-H.L.); hzhou@must.edu.mo (H.Z.); 2 School of Chinese Medicines, Macau University of Science and Technology, Avenida Wai Long, Taipa, Macao, China; 1209853gc211002@student.must.edu.mo; 3 International Institute for Translational Chinese Medicine, Guangzhou University of Chinese Medicine, Guangzhou 510006, China; liuzqsmu@gmail.com; 4 School of Basic Medicinal Sciences and Nursing, Chengdu University, Chengdu 610106, China; yingliu_1971@163.com

**Keywords:** *Aconitum carmichaelii*, lipo-alkaloids, C19-aconitane skeletons, oxygenated fatty acids, UHPLC-Q-TOF-MS

## Abstract

Lipo-alkaloid is a kind of C19-norditerpenoid alkaloid usually found in *Aconitum* species. Structurally, they contain an aconitane skeleton and one or two fatty acid moieties of 3–25 carbon chains with 1–6 unsaturated degrees. Analysis of the lipo-alkaloids in roots of *Aconitum carmichaelii* resulted in the isolation of six known pure lipo-alkaloids (**A1**–**A6**) and a lipo-alkaloid mixture (**A7**). The mixture shared the same aconitane skeleton of 14-benzoylmesaconine, but their side chains were determined to be 9-hydroxy-octadecadienoic acid, 13-hydroxy-octadecadienoic acid and 10-hydroxy-octadecadienoic acid, respectively, by MS/MS analysis after alkaline hydrolysis. To our knowledge, this is the first time of the reporting of the oxygenated fatty acids as the side chains in naturally-occurring lipo-alkaloids. In order to identify more lipo-alkaloids, a compound database was established based on various combinations between the aconitane skeleton and the fatty acid chain, and then, the identification of lipo-alkaloids was conducted using the database, UHPLC-Q-TOF-MS and MS/MS. Finally, 148 lipo-alkaloids were identified from *A. carmichaelii* after intensive MS/MS analysis, including 93 potential new compounds and 38 compounds with oxygenated fatty acid moieties.

## 1. Introduction

Lipo-alkaloid is a kind of C19-norditerpenoid alkaloid usually found in *Aconitum* species. Structurally, they consist of an aconitane skeleton and one or two fatty acid moieties of 3–25 carbon chains with 1–6 unsaturated degrees [1]. So far, more than 200 lipo-alkaloids have been reported from plants [1], semisynthesis [2] and biotransformations [3,4,5]. Because naturally-occurring lipo-alkaloids are so close structurally, it is very difficult to purify them from the mixture. Their characterization is mainly conducted by high sensitive mass spectrometry (MS) or liquid chromatography-mass spectrometry (LC-MS) techniques, such as ion trap-MS [6], MALDI-TOF-MS [7], ESI-Fourier Transform Ion Cyclotron Resonance FTICR-MS [8], LC-ESI-Ion TrapIT-MS [8,9,10], and LC-Linear Ion TrapLTQ-Orbitrap-MS [11]. To our knowledge, only one pure lipo-alkaloid, 8-*O*-azeloyl-14-benzoylaconine, was isolated and elucidated by NMR from plants [12]. In this research, the separation of naturally-occurring lipo-alkaloids was optimized using column chromatography (CC), and six pure lipo-alkaloids (**A1**–**A6**) were obtained from a natural source for the first time. At the same time, a mixture of oxygenated fatty acid-containing lipo-alkaloids (**A7**) was also obtained, and the structures were determined by MS/MS analysis after alkaline hydrolysis. Furthermore, a compound database of lipo-alkaloids was established based on the basic skeletons and fatty acids groups, and by the combination of the compound database, UHPLC-Q-TOF-MS and MS/MS analysis, 148 lipo-alkaloids, including 93 potential new ones, were identified from *A. carmichaelii*.

## 2. Results and Discussion

### 2.1. Isolation and Structural Elucidation of Lipo-Alkaloids ***A1**–**A7***

**Figure 1:**
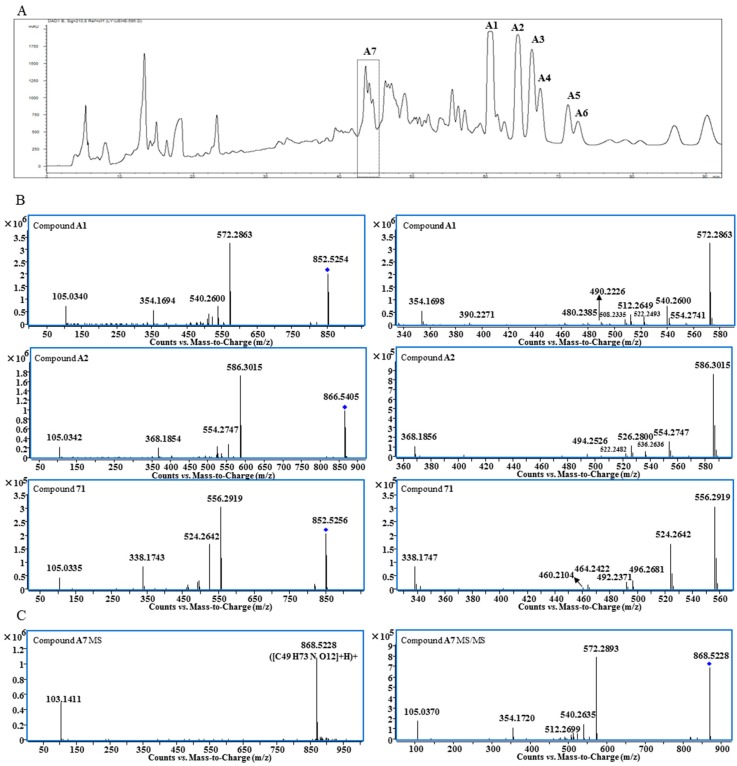
Preparative HPLC chromatogram of Fraction **D4-1-8** (**A**); MS/MS and expanded MS/MS spectra of Compounds **A1**, **A2** and **71** (**B**); MS and MS/MS spectra of Compound **A7** (**C**).

Structurally, lipo-alkaloids usually contain one or two long fatty acid moieties; therefore, they have low polarity and can be extracted by *n*-hexane. After column chromatography separation on silica gel and ODS, a lipo-alkaloids-rich fraction was obtained. Compared to water and methanol as mobile phases, 0.01% diethylamine in water and methanol gave better separation of lipo-alkaloids on a preparative HPLC C18 column (Figure 1A). Seven compounds (**A1**–**A7**) were determined to be lipo-alkaloids by UHPLC-Q-TOF-MS and NMR techniques.

The molecular formula of Compound **A1** was determined as C_49_H_73_NO_11_ based on the quasi-molecular ion at *m*/*z* 852.5254. The base peak at *m*/*z* 572.2863 in the MS/MS spectrum is produced from the neutral loss of C_18_H_32_O_2_ (Figure 1B), indicating the presence of long chain fatty acid moiety. Other fragmentation ions at *m*/*z* 540.2600, 522.2493, 512.2649, 508.2335, 490.2226, 480.2385 and 390.2271 were related to the neutral losses of MeOH, H_2_O, CO and BzOH (Table 1), while the fragmentation ion at *m*/*z* 105.0340 was assigned to the benzoyl group. Therefore, the basic skeleton of **A1** should be 14-benzoylmesaconitine (BMA) [13], and this was supported by its ^1^H- and ^13^C-NMR spectra (Table S1). ^1^H-NMR also displayed the unconjugated vinylic protons at δ 5.376 (4H, m, H-9″, 10″, 12″, 13″), bis-allylic protons at δ 2.802 (2H, m, H-11″), allylic protons at δ 2.045 (4H, m, H-8″, 14″), acylated methylene at δ 1.450 (1H, m, H-2″a) and 1.816 (1H, m, H-2″b), methylene at δ 1.380 (8H, m, H-7″, 15″, 16″, 17″), 1.177 (2H, m, H-6″), 1.026 (2H, m, H-5″), 1.179 (1H, m, H-3″a), 1.025 (1H, m, H-3″b) and 0.885 (2H, m, H-4″) and a methyl group at δ 0.915 (3H, t, H-18″); thus, the fatty acid moiety was determined to be the linoleoyl group. Comparing to the ^1^H-NMR spectra of free fatty acids, significant up-field shifting was observed for H-2″, H-3″ and H-4″; moreover, the signals of two protons of the methylene at positions 2″ and 3″ were un-equivalent, which should be induced by the 14-benzoyl group. Finally, Compound **A1** was determined to be 8-*O*-linoleoyl-14-benzoylmesaconine.

The molecular formulae of Compounds **A3** and **A4** were C_47_H_73_NO_11_ and C_49_H_75_NO_11_, which were two carbons less and two hydrogens more than that of Compound **A1**. The same MS/MS spectra (Table S2) as **A1** indicated that **A3** and **A4** should be BMA derivatives. Meanwhile, the neutral losses of C_16_H_32_O_2_ and C_18_H_34_O_2_ in MS/MS spectra and ^1^H-NMR data (Table S1) suggested that the side chains for **A3** and **A4** were palmitic acid and oleic acid, respectively. Therefore, their structures were determined to be 8-*O*-palmitoyl-14-benzoylmesaconine (**A3**) and 8-*O*-oleoyl-14-benzoylmesaconine (**A4**).

The molecular formula of Peak **A2** was determined as C_50_H_75_NO_11_ from the high resolution MS, which was one carbon and two hydrogens more than that of **A1**. The pattern of fragmentation ions of **A2** was very similar to that of **A1** with most of the peaks moved 14 Da (CH_2_) to the right side (Figure 1B and Table 1). The ^1^H-NMR spectrum of **A2** showed the presence of *N*CH_2_CH_3_ (Table S1); therefore, the basic skeleton should be 14-benzoylaconine (BA), and it was determined to be 8-*O*-linoleoyl-14-benzoylaconine. With a similar comparison, Compounds **A5** (C_48_H_75_NO_11_) and **A6** (C_50_H_77_NO_11_) were determined to be 8-*O*-palmitoyl-14-benzoylaconine and 8-*O*-oleoyl-14-benzoylaconine, respectively, based on their MS/MS and ^1^H-NMR spectra.

**Figure 2:**
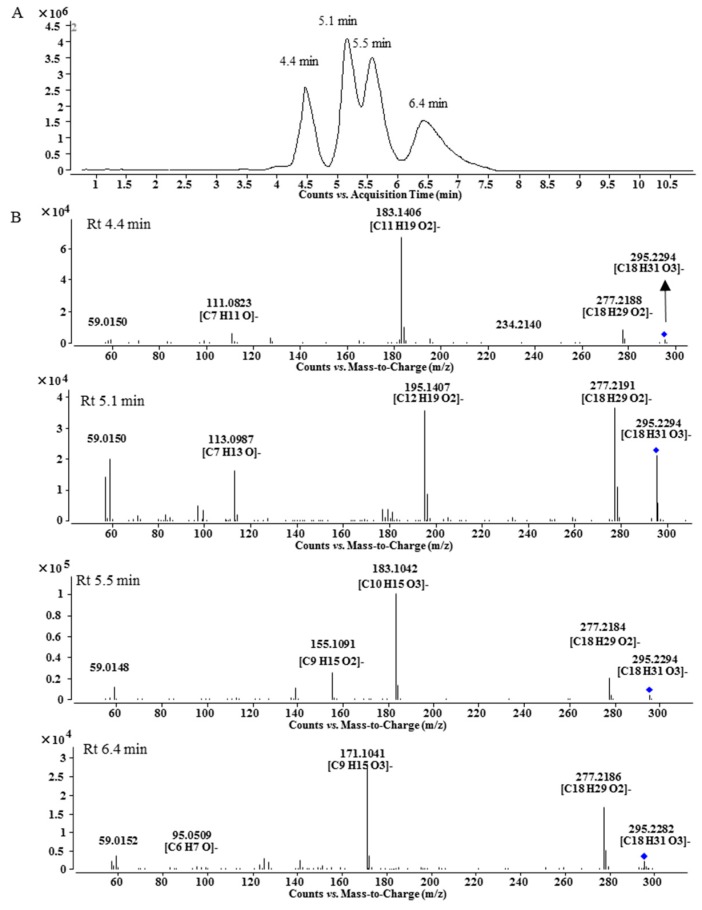
LC-MS chromatogram (**A**) and MS/MS spectra (**B**) of fatty acids released by alkaline hydrolysis.

Peak **A7** was obtained as a single peak in the UHPLC-MS chromatogram and with a quasi-molecular ion at *m*/*z* 868.5228, which is in agreement with the molecular formula of C_49_H_73_NO_12_ that is one oxygen more than that of **A1**. The MS/MS spectrum showed that **A7** was a BMA derivative with C_18_H_32_O_3_ as the side chain (Figure 1C). However, the ^1^H-NMR spectrum of **A7** was unexpectedly complicated. Except for BMA signals, the unconjugated vinyl protons at δ 5.371 (4H, m), bis-allylic methylene at δ 2.790, allylic methylene at *δ* 2.051 and methylene at around *δ* 1.3, there were three proton signals at δ 4.413, 4.189 and 4.127 with unproportioned integrations to other signals. Therefore, **A7** might be a mixture containing different side chains. After alkaline hydrolysis of **A7**, the released fatty acids were analyzed by UHPLC-MS and MS/MS, and the results are shown in Figure 2. At least four peaks were detected at 4.4, 5.1, 5.5 and 6.4 min with the same *m*/*z* at 295.23 (C_18_H_32_O_3_), but their MS/MS spectra were significantly different (Figure 2B). In the MS/MS spectrum of the peak at 5.1 min, the fragmentation ions at *m*/*z* 277.2191 ([M − H − H_2_O]^−^) and 195.1407 ([M − H − C_6_H_12_O]^−^) indicate the presence of a hydroxyl group at C-13 [14] and the unconjugated vinyl bonds should be between C-2 and C-12, *i.e.*, 13-hydroxyoctadecadienoic acid. The fragmentation ions in the MS/MS spectrum for the third peak at 5.5 min were related to the neutral losses of H_2_O, C_8_H_16_ and CO, just like the MS/MS spectrum of 10-hydroxyoctadecadienoic acid [15]. The fourth peak was identified as 9-hydroxyoctadecadienoic acid based on the ions of [M − H − H_2_O]^−^ and [M − H − C_9_H_16_]^−^ [14]. For the first peak, the fragmentation ions from the neutral losses of H_2_O and C_5_H_12_O were observed. The cleavage of hydroxyl group usually gives an unsaturated carbonyl group, which is different from the neutral loss of C_5_H_12_O, so the fatty acid at 4.4 min needs further investigation. Finally, Peak **A7** was determined as the mixture of the oxygenated fatty acids-containing BMA derivative by the combination of ^1^H-NMR and MS/MS analysis after alkaline hydrolysis.

**Figure 3:**
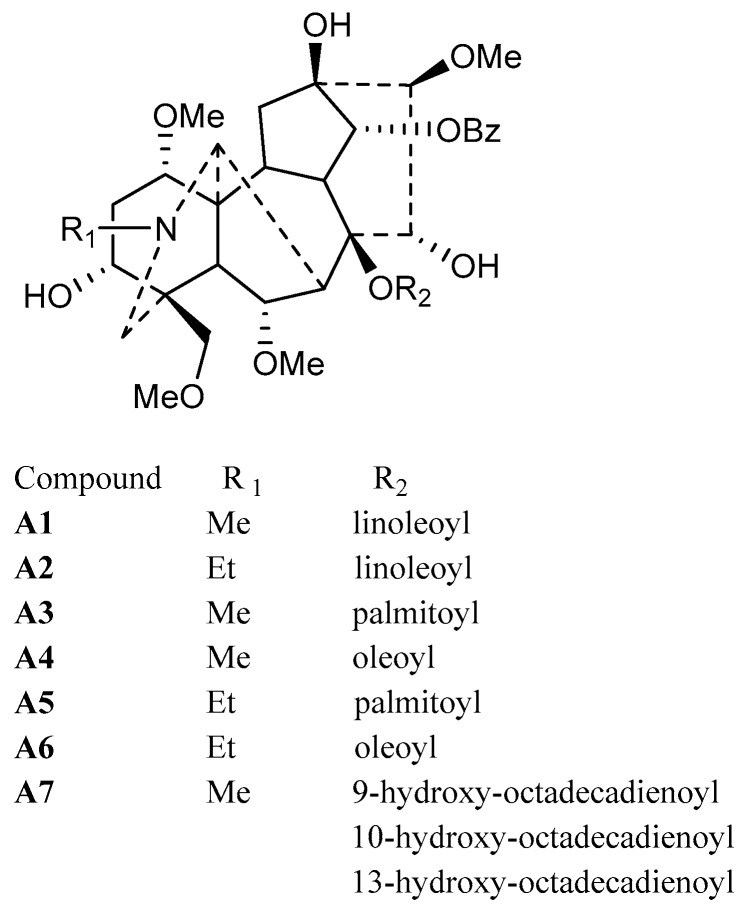
Structures of isolated lipo-alkaloids (**A1**–**A7**).

As a result, six pure lipo-alkaloids were obtained by optimized column chromatography and characterized as 8-*O*-linoleoyl-14-benzoylmesaconine (**A1**), 8-*O*-linoleoyl-14-benzoylaconine (**A2**), 8-*O*-palmitoyl-14-benzoylmesaconine (**A3**), 8-*O*-oleoyl-14-benzoylmesaconine (**A4**), 8-*O*-palmitoyl-14-benzoylaconine (**A5**) and 8-*O*-oleoyl-14-benzoylaconine (**A6**), respectively. Although these compounds were identified by LC-MS and/or semi-synthetic methods before, this is the first time that they have been purified from nature. Besides, oxygenated fatty acid-containing lipo-alkaloids were firstly obtained, and they shared the same aconitane skeleton of 14-benzoylmesaconine, while their side chains were determined to be 9-hydroxy-octadecadienoic acid, 13-hydroxy-octadecadienoic acid and 10-hydroxy-octadecadienoic acid, respectively. Their structures are shown in Figure 3.

### 2.2. Establishment of Lipo-Alkaloids Database

In the structures of the previously-reported naturally-occurring lipo-alkaloids [1,11], there were 13 basic aconitine skeletons and 51 fatty acid chains. Oxygenated fatty acids are widely present in plants; thus, it is possible that these fatty acids connect with aconitane skeletons to form the lipo-alkaloids, as reported above. All possible lipo-alkaloids can be hypothesized by the following formula:

MF_lipo-alkaloid_ = MF_basic skeleton_ + MF_fatty acid_ − H_2_O (1)

Herein, MF_lipo-alkaloid_ and MF_basic skeleton_ are the molecular formulae of potential lipo-alkaloids and the 13 reported aconitane skeletons, while MF_fatty acid_ is the molecular formula of possible fatty acids, which have 3–25 carbons and 2–6 oxygen atoms with the unsaturated degrees from 1–7. Then, the names and molecular formulae of the hypothesized lipo-alkaloids were input into an Agilent MassHunter database file to establish an in-house lipo-alkaloids database with a total of 484 molecular formulae.

### 2.3. Determination of Lipo-Alkaloids by Combination of Database, UHPLC-Q-TOF-MS and MS/MS Analysis

**Figure 4:**
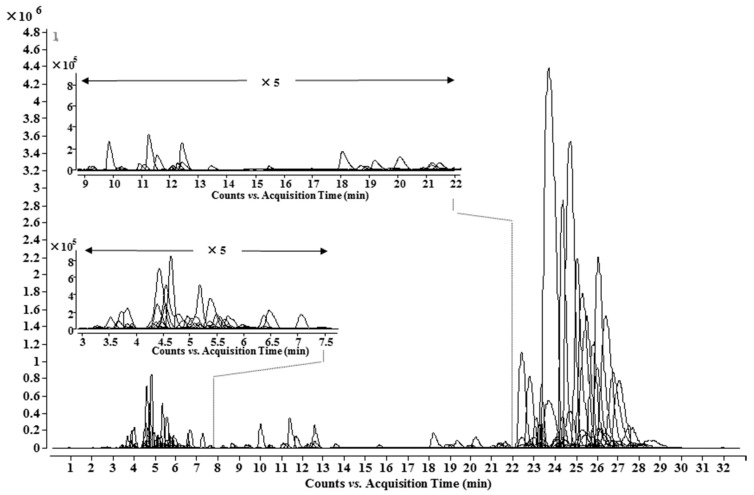
UHPLC-MS chromatogram of the identified lipo-alkaloids in *A. carmichaelii*.

Based on information from the lipo-alkaloids database, UHPLC-Q-TOF-MS and MS/MS analysis, 148 lipo-alkaloids, including 93 potential new ones, were determined (Table 2 and Figure 4). Among them, 21 molecular formulae were found to have at least two isomers with different MS/MS spectra and/or retention times, e.g., Compounds **71** and **102**, **79** and **122**, **62**, **72** and **109**. Compounds **71** and **102** had the same molecular formula of C_49_H_73_NO_11_. Compound **102** has the same retention time, MS and MS/MS spectra as Compound **A1**, 8-*O*-linoleoyl-14-benzoylmesaconine. The MS/MS base peak of **71** was at *m*/*z* 556.2919, which was one oxygen less than that of **A1**; other fragmentation ions were also one oxygen less than the corresponding ions in **A1**, and no ion related to neutral loss of CO was observed (Table 1). Therefore, the basic skeleton of **71** was identified as 14-benzyolhypaconine (BHA). The fatty acid in **71** was determined to have a formula of C_18_H_32_O_3_, corresponding to a hydroxyoctadecadienoic acid as in Compound **A7**. Similarly, Compounds **79** and **122** have the same molecular formula of C_49_H_75_NO_11_ with two more hydrogens than **71** and **A1**. Compound **122** was unambiguously determined as 8-*O*-oleoyl-14-benzoylmesaconine based on the same retention time, MS and MS/MS spectra as **A4**. The MS/MS spectrum of **79** suggested a basic skeleton of BHA, so the fatty acid should have two hydrogens more than that in **71**, *i.e.*, hydroxyoctadecenoic acid (C_18_H_34_O_3_) in **79**. Compounds **61**, **72** and **109** had one oxygen more than **79** and **A4**, and the fragmentation patterns of **61** and **72** were almost the same as those of **79** and **A4**; thus, the additional oxygen should substitute on the fatty acid side chains, which are C_18_H_34_O_4_ and C_18_H_34_O_3_, respectively. The MS/MS base peak ion of Compound **109** showed one oxygen more than that of **72**, and other fragmentation ions were also 16 Da more than that of Compound **72**; therefore, the basic skeleton should be 10-OH-BMA. Therefore, Compound **109** was identified as 8-*O*-oleoyl-10-hydroxy-14-benzoylmesaconine. Similarly, by comprehensive analysis of the MS/MS spectra, 22 other fatty acid chains, e.g., C_18_H_30_O_3_, C_18_H_32_O_4_, C_18_H_36_O_3_ and C_23_H_46_O_2_, were also determined, as shown in Table 3.

**Figure 5:**
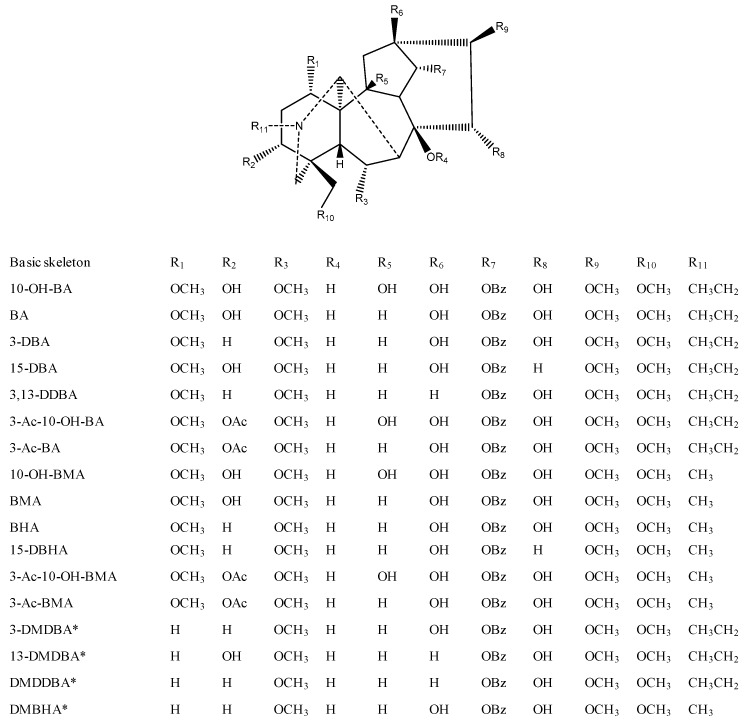
Structures of aconitane skeletons of lipo-alkaloids. * Indicates the skeleton was detected for the first time.

**Figure 6:**
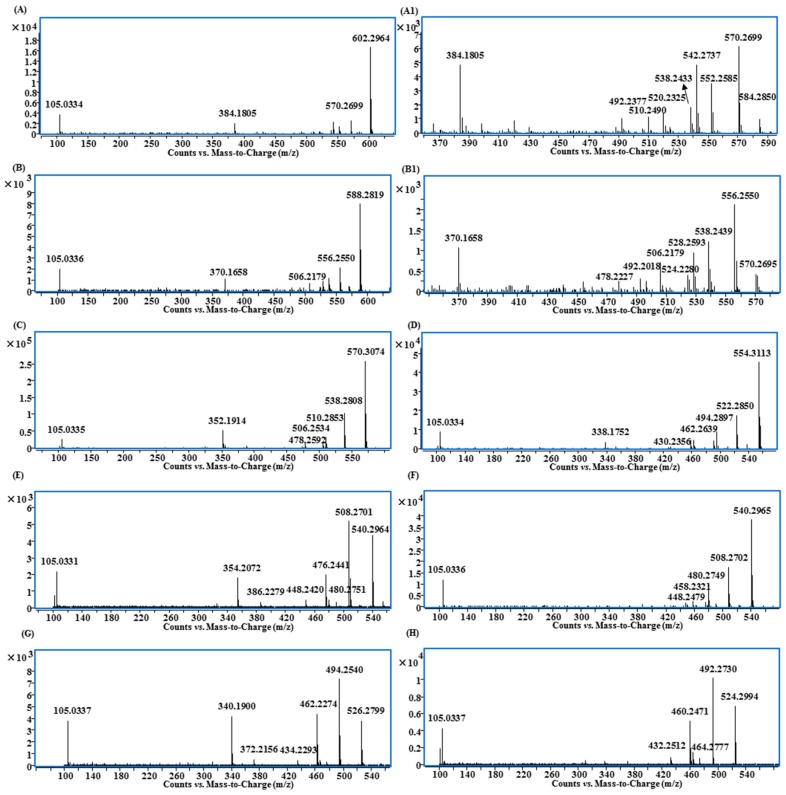
MS/MS spectra of lipo-alkaloids with the basic skeletons of: (**A and A1**) 10-OH-BA; (**B and B1**) 10-OH-BMA; (**C**) 3-DBA; (**D**) 3,13-DDBA; (**E**) 3-DMDBA; (**F**) 13-DMDBA; (**G**) DMBHA; (**H**) DMDDBA.

In addition to 13 known aconitane skeletons included in the database, four new aconitane skeletons were found in the identified lipo-alkaloids for the first time (Figure 5). The elucidation of new skeletons is discussed below, while the MS/MS characterization of known skeletons is shown in Figure 6 and Table 1.

Compounds **85**, **103**, **116** and **117** shared the same fragmentation patterns, in which *m*/*z* 540.30 ([M + H − FA]^+^), 508.27 ([M + H − FA − CH_3_OH]^+^), 480.28 ([M + H − FA − CH_3_OH − CO]^+^), 476.24 ([M + H − FA − 2CH_3_OH]^+^), 448.24 ([M + H − FA − 2CH_3_OH − CO]^+^), 354.21 ([M + H − FA − 2CH_3_OH − benzoic acid]^+^) and 105.03 ([C_6_H_5_CO]^+^) were the major ions (Figure 6E). Except for the ion at *m*/*z* 354.21, all other ions contained one OCH_2_ group less than that of 3-deoxy-14-benzoylaconine (3-DBA) derivatives (Figure 6C); therefore the basic skeleton was determined to be demethoxy-3-deoxy-14-benzoylaconine (3-DMDBA). The ion at *m*/*z* 354.21 was derived from the loss of two methanols rather than three methanols, which in turn further confirmed that the basic skeleton had one methoxyl group less than 3-DBA with 1-OCH_3_ or 6-OCH_3_ missing (Scheme S4).

The isomers of Compounds **103**, **117** and **116** were observed at the retention times of 24.5 min (**97**), 26.0 (**115**) and 26.1 min (**119**), respectively. They had similar MS/MS fragmentation ions, which were significantly different from Compounds **103**, **117** and **116**. The ions of [M + H − FA]^+^, [M + H − FA − CH_3_OH]^+^, [M + H − FA − CH_3_OH − CO]^+^, [M + H − FA − 2CH_3_OH]^+^, [M + H − FA − 2CH_3_OH − CO]^+^ and [C_6_H_5_CO]^+^ were also observed, but the relative intensities of [M + H − FA]^+^ and [M + H − FA − CH_3_OH − CO]^+^ were higher than others (Figure 6F). More importantly, there was no evidence of the losses of FA + 2CH_3_OH + benzoic acid, so that the basic skeleton should be demethoxy-13-deoxy-14-benzoylaconine (13-DMDBA).

Compounds **51**, **95** and **110** had the molecular formulae of C_46_H_71_NO_9_, C_48_H_71_NO_9_ and C_48_H_73_NO_9_, and their fragmentation ions were mainly generated from the neutral losses of FA, methanol and benzoic acid showing at *m*/*z* 526.28, 494.25, 462.23 and 340.19 (Figure 6G and Scheme S3). The fragmentation patterns were very similar to those of 3-DMDBA derivatives (Figure 6E), but with one CH_2_ less. The ions corresponding to the loss of two molecules of methanol at *m*/*z* 462.23 ([M + H − FA − 2CH_3_OH]^+^) and 340.19 ([M + H − FA − 2CH_3_OH − benzoic acid]^+^) indicated that the differences were the substitution groups on the *N* atom, and it should be *N*-CH_3_ rather than *N*-C_2_H_5_ in these three compounds, *i.e.*, the basic skeleton should be demethoxy-14-benzoylhypaconine (DMBHA).

Compounds **105**, **120**, **131** and **137** shared the same fragment ions at *m*/*z* 524.30, 492.27, 464.28, 460.25 and 432.25 (Figure 6H). The fragmentation patterns were very similar to those compounds with 3-DMDBA as the basic skeleton (Figure 6E), but with one oxygen less. Because there was no ion produced from the loss of FA + methanol + benzoic acid, the absence of 13-OH was indicated; therefore, the basic skeleton should be demethoxy-3,13-dideoxy-14-benzoylaconine (DMDDBA).

Based on the finding of oxygenated fatty acids as the side chains of lipo-alkaloids, the possible lipo-alkaloids were predicted and included in an in-house database. By the combination of the database, UHPLC-MS and MS/MS analysis, not only more oxygenated fatty acid-containing lipo-alkaloids were determined, but also four aconitane skeletons not reported in lipo-alkaloids before were detected. Finally, 148 lipo-alkaloids, including 93 potential new ones, were identified (Table 2). Although most of previous reports showed that the contents of lipo-alkaloids usually increased after processing, no significant difference was detected when using heat reflux extraction or ultrasonic extraction in our preliminary research (data not shown).

### 2.4. MS/MS Characterizations of Aconitane Skeletons in Lipo-Alkaloids

In this study, we reported 13 aconitane skeletons (including four new ones) in the lipo-alkaloids with their main fragmentation ions from the neutral losses of MeOH, H_2_O, CO and BzOH (Figure 6 and Table 1). Based on structures and MS/MS spectra, the relationship between the substitutions and the fragmentation ions can be summarized as follows. (1) The ions produced from the neutral loss of MeOH have higher abundance, and the numbers of methoxy group substituted on aconitane skeleton usually are determined from the corresponding ions. For instance, ions with the loss of three molecules of methanol were detected for the aconitine skeletons with tetramethoxy substitution, while ions with neutral loss of two molecules of methanol were observed for the trimethoxy-substituted skeletons. Due to the higher bond energy between C_18_ and the methoxy group [22], it is difficult to detect the fragment ions from the loss of C_18_-OMe; (2) The ion corresponding to the neutral loss of BzOH should be a diagnostic ion of 13-OH-14-OBz. The ion of [M + H − FA − 3CH_3_OH − BzOH]^+^ was observed for the lipo-alkaloids with the basic skeletons of 3-Ac-BMA, 10-OH-BA, 10-OH-MA, BA, BMA, DBA and BHA, and the ion of [M + H-FA-2CH_3_OH-BzOH]^+^ was detected from the derivatives of 3-DMDBA and DMBHA, while no evident ion was found in MS/MS spectra of the 3,13-DDBA, 13-DMDBA and 3,13-DMDDBA derivatives (Table 1). Moreover, the aforementioned ion could also be used to determine the numbers of methoxy groups substituted on the aconitane skeletons; (3) The ions of the loss of CO indicates the presence of the 15-OH-16-OMe group [11], e.g., [M + H − FA − 2CH_3_OH − CO]^+^ was observed in all identified lipo-alkaloids. (4) The loss of H_2_O usually indicates the substitution of a hydroxyl group at C-3. There is no such ion observed in DBA, BHA, 3, 13-DDBA, 3-DMDBA, DMBHA and 3, 13-DMDDBA.

### 2.5. Fatty Acid Side Chains in Lipo-Alkaloids

Besides common long chain fatty acids, medium and long chain oxidized fatty acids were detected as the side chains of lipo-alkaloids in plants for the first time, e.g., C_9_H_16_O_3_, C_18_H_30_O_3_, C_18_H_32_O_3_, C_18_H_32_O_4_, C_18_H_34_O_3_, C_18_H_34_O_4_, C_18_H_34_O_5_, and so on (Table 3). These oxygenated fatty acids might occur as hydroxyl-, oxo-, epoxy-, hydroperoxy-type or diacid [23]. However, due to the limitation of LC-MS data, it is difficult to determine in which form they exist in the lipo-alkaloids. In this study, three oxygenated fatty acids in a lipo-alkaloids mixture were determined by ^1^H-NMR, alkaline hydrolysis and MS/MS analysis, but other oxygenated fatty acid groups could not be determined due to the limited amount of sample available. Considering the polarity and occurrence of fatty acids in nature, the most possible structures were proposed in Table 2 by searching the lipid maps [24] and comparing retention times of the lipo-alkaloids to common fatty acid side chains.

Plant oxylipins are involved in the stress responses, and some of them have anti-microbial and anti-insecticidal activities [25]. Some oxylipins, e.g., 2-hydroxyoleic acid (C_18_H_34_O_3_), were found to have anti-cancer activity [26], while some oxylipins have anti-inflammatory activity [27]. When these oxidized fatty acids connect to aconitane alkaloids to form the lipo-alkaloids, the bioactivity and toxicity of aconitane alkaloids might change. Thus, the occurrence, bioactivity and toxicity of these oxygenated fatty acid-containing lipo-alkaloids are worth further investigations.

## 3. Materials and Methods

### 3.1. Chemicals and Reagents

AR-grade *n*-hexane, dichloromethane, *n*-butanol, methanol and HPLC-grade methanol were obtained from Anaqua Chemicals Supply (Houston, TX, USA). MS-grade acetonitrile, methanol and water were purchased from J.T. Baker (Danville, PA, USA), and MS-grade formic acid was provided by Sigma-Aldrich Laboratories, Inc. (St. Louis, MO, USA). AR-grade potassium hydroxide, hydrochloric acid (37%), and diethylamine were purchased from Merck and Advanced Technology & Industrial CO. Ltd. (Hong Kong, China), respectively. Silica gel (75–150 mesh) and ODS (35–70 µm) were provided by Grace (Columbia, MD, USA).

### 3.2. Plant Materials

The roots of *Aconitum carmichaelii* Debx. (ChW-02) were obtained from Hehuachi Medicinal Materials Market in Chengdu, Sichuan Province of China, and authenticated by Ying Liu, Chengdu University. A voucher specimen was deposited in Macau University of Science and Technology.

### 3.3. Separation of Lipo-Alkaloids

The air-dried roots of *A. carmichaelii* (7.2 kg) were powdered and soaked in methanol (12 L) at room temperature for one week and then extracted with methanol at reflux 3 times (3 × 12 L, 1 h for each extraction). The combined methanol extracts were evaporated under vacuum to give 356 g of residue, which was suspended in distilled water (3 L) followed by the participation with *n*-hexane (3 × 3 L), ethyl acetate (3 × 3 L) and *n*-butanol (3 × 3 L), successively. The *n*-hexane extract (32 g) was subjected to silica gel CC (6 × 60 cm) using CH_2_Cl_2_/CH_3_OH as the eluate to provide four fractions (A–D). Fraction D (5 g) was further subjected to ODS CC (4.5 × 50 cm) using water-containing methanol (0%–100%) as the eluate to produce five subfractions (D1–5). Subfraction D4 (1 g) was divided into 10 parts by another ODS CC with an increasing gradient of water-containing methanol (40%–100%). Preparative HPLC separation of the eighth part (D4–8, 134 mg) on an ODS column (10 × 250 mm, 5 µm) produced 7 compounds, 8 mg **A1**, 6 mg **A2**, 3 mg **A3**, 9 mg **A4**, 7 mg **A5**, 3 mg **A6** and 4 mg **A7**. The mobile phases were 0.01% diethylamine-containing water (A) and methanol (B) with the following gradient: 0–40 min, 70%–95 B%; 40–120 min, 95% B. The flow rate was 2 mL/min, and the detection wavelength was set at 230 nm. The structures were characterized by NMR and mass spectrometry.

### 3.4. Alkaline Hydrolysis of Peak ***A7***

One milligram of A7 was dissolved in 400 µL of KOH-saturated methanol solution and then heated to 75 °C for 15 min and 60 min. The reaction solution was neutralized with 800 µL of 5 M HCl-MeOH and participated with ethyl acetate, respectively. The ethyl acetate layer was analyzed by UHPLC-Q-TOF-MS.

### 3.5. Preparation of Methanol Extracts of Herbal Sample

One gram of powdered herbal sample was extracted with 6 mL methanol for 60 min with the aid of an ultrasonicator and then centrifuged at 13,000 rpm for 10 min. The supernatant was collected and diluted 10-times, then followed by the acquisition of UHPLC-Q-TOF-MS data.

### 3.6. UHPLC-Q-TOF-MS Analysis

Agilent 1290 UHPLC system (UHPLC, Agilent Technologies, Santa Clara, CA, USA) consisting of an autosampler, thermostated column compartment and binary pump and equipped with an Agilent Eclipse C18 column (2.1 × 100 mm, 1.8 μm, Agilent Technologies) was applied for the separation of components. The mobile phases were 0.1% formic acid in water (A) and 0.1% formic acid in acetonitrile (B). Method 1 was applied for the determination of lipo-alkaloids, and the mobile phase gradient was set as follows, 0–0.5 min, 20% B; 0.5–30 min, 20%–98% B; 30–33 min 98% B; 33–33.1 min, 98%–20% B, and then maintained for 2 min. Method 2 was used for the analysis of fatty acids, and the gradient was 0–11 min, 25% B, 11–11.1 min, 25%–95% B, and then maintained for 2 min. The flow rate was 0.3 mL/min, and the injection volume was 2 µL. The mass spectrometry was conducted on a 6550 UHD Accurate-Mass Q-TOF/MS system (Agilent Technologies) with a dual Agilent Jet Stream electrospray ion source (dual AJS ESI). The mass parameters were optimized using the standards of aconitine, mesaconitine and hypaconitine and set as follows: dry gas temperature and flow were 250 °C and 15 L/min; sheath gas temperature and flow were 300 °C and 11 L/min; nebulizer at 20 psi; the capillary and nozzle voltages were 4000 and 500 V, respectively. The fragmentor was 380 V, and the collision cell energies were set at 50 eV for lipo-alkaloids in positive mode and 30 eV for fatty acids in negative mode, respectively.

### 3.7. Establishment of the Lipo-Alkaloids Database

Based on the possible fatty acid chains and known aconitane skeletons reported in *Aconitum* plants, the possible lipo-alkaloids were hypothesized and input into Agilent MassHunter database file (“Compound Formula Database”) to establish an in-house lipo-alkaloids database. Then, the potential lipo-alkaloids in *A. carmichaelii* were extracted using the function of “Find Compounds by Formula (FBF)” and determined by MS/MS analysis.

## 4. Conclusions

In this study, the separation method of lipo-alkaloids was optimized, and using this method, oxygenated fatty acids-containing lipo-alkaloids were obtained for the first time. A lipo-alkaloids database was established based on the known basic aconitane skeletons and possible fatty acid side chains. By using the database, potential lipo-alkaloids were first extracted from UHPLC-Q-TOF-MS, and then, the structures were determined from the comprehensive analysis and deduction of MS/MS spectra, resulting in successful identification of 148 lipo-alkaloids. Among them, 38 compounds contain medium or long chain oxidized fatty acids as side chains that were not reported previously. The combination of database and LC-MS dramatically speeds up the finding of potential new compounds and is confirmed to be a powerful tool in the study of natural product chemistry. The new finding of oxygenated fatty acids as side chains of lipo-alkaloids provides a kind of possible structures, which accounts for the bioactivities of *A. carmichaelii*, a widely-used traditional medicine.

## Figures and Tables

**Table 1:** MS/MS characteristic fragmentation ions of aconitane skeletons in lipo-alkaloids ^a^.

Basic Skeleton	[M + H − FA]^+^	[M + H − FA − CH_3_OH]^+^	[M + H − FA − CH_3_OH − H_2_O]^+^	[M + H − FA − CH_3_OH − CO]^+^	[M + H − FA − 2CH_3_OH]^+^	[M + H − FA − 2CH_3_OH − H_2_O]^+^	[M + H − FA − 2CH_3_OH − CO]^+^	[M + H − FA − 3CH_3_OH]^+^	[M + H − FA − 2CH_3_OH − BzOH]^+^	[M + H − FA − 3CH_3_OH − BzOH]^+^	[M + H − FA − 3CH_3_OH − BzOH − AcOH]^+^
3-Ac-BMA	614.2965	582.2703	-	554.2754	550.2441	-	522.2492	-	-	396.1811	336.1600
10-OH-BA	602.2965	570.2703	552.2597	542.2754	538.2441	520.2335	510.2492	506.2179	weak	384.1811	-
10-OH-BMA	588.2809	556.2547	538.2441	528.2597	524.2284	506.2179	496.2335	492.2022	weak	370.1654	-
BA	586.3016	554.2754	536.2648	526.2805	522.2492	504.2386	494.2543	490.2230	weak	368.1862	-
BMA	572.2860	540.2597	522.2492	512.2648	508.2335	490.2230	480.2386	476.2073	weak	354.1705	-
DBA	570.3067	538.2805	-	510.2856	506.2543	-	478.2593	-	weak	352.1913	-
BHA	556.2910	524.2648	-	496.2704	492.2386	-	464.2437	460.2124	weak	338.1756	-
3,13-DDBA	554.3118	522.2856	-	494.2906	490.2593	-	462.2644	458.2331	-	-	-
3-DMDBA *	540.2961	508.2699	-	480.2750	476.2437	-	448.2448	-	354.2069	-	-
13-DMDBA *	540.2961	508.2699	490.2593	480.2750	476.2437	458.2331	448.2448	-	-	-	-
DMBHA *	526.2805	494.2543	-	-	462.2280	-	434.2331	-	340.1913	-	-
3,13-DMDDBA *	524.3014	492.2750	-	464.2801	460.2488	-	432.2539	-	-	-	-

^a^ The *m*/*z* of fragmentation ions is shown as calculated values. * New skeleton found in lipo-alkaloid. FA: fatty acid.

**Table 2:** Lipo-alkaloids identified from *A. carmichaelii*.

No.	Rt (min)	Calcd. [M + H]^+^	[M + H]^+^	Alkaloids	Ref.	No.	Rt (min)	Calcd. [M + H]^+^	[M + H]^+^	Alkaloids	Ref.
1	3.4	692.3277	692.3285	8-dhbtn-BMA *		75	21.8	884.5519	884.5494	8-hode-BA *	
2	3.4	762.2968	762.2968	8-act-10-OH-BMA *		76	22.4	866.5049	866.5061	8-linolen-10-OH-BMA	[16]
3	3.6	902.3805	902.3777	8-gvl-BMA *		77	22.5	880.5206	880.5193	8-linolen-10-OH-BA	[16]
4	3.8	746.3018	746.3002	8-act-BMA *		78	22.5	824.4943	824.4888	8-pmde-BMA	[8]
5	4.0	692.3277	692.3258	8-hbtn-10-OH-BMA *		79	22.7	854.5413	854.5409	8-hode-BHA	[8]
6	4.2	914.3805	914.3784	8-gcf-BMA *		80	22.9	850.5100	850.5105	8-linolen-BMA	[6]
7	4.5	928.3961	928.3946	8-gfr-BMA *		81	22.9	812.4943	812.4874	8-ptde-BMA	[6]
8	4.6	730.3069	730.3041	8-act-BHA *		82	23.0	772.4630	772.4618	8-laur-BMA *	
9	4.7	734.3382	734.3324	8-adp-10-OH-BMA *		83	23.1	864.5256	864.5252	8-linolen-BA	[8]
10	4.7	734.3382	734.3324	8-hadp-BMA *		84	23.2	826.5100	826.5052	8-pme-BMA	[7]
11	4.8	676.3328	676.3321	8-hbtn-BMA *		85	23.3	818.5202	818.5209	8-linolen-3-DMDBA *	
12	5.0	760.3175	760.3189	8-act-BA *		86	23.4	848.5307	848.5310	8-linolen-DBA	[6]
13	5.3	760.3175	760.3176	8-dohpnd-BMA *		87	23.6	868.5206	868.5195	8-lino-10-OH-BMA	[17]
14	5.6	672.3015	672.3020	8-fmr-BHA *		88	23.8	838.5100	838.5105	8-hpdde-BMA *	
15	5.8	660.3384	660.3380	8-hbtn-BHA *		89	23.8	840.5256	840.5184	8-pme-BA	[18]
16	5.8	744.3226	744.3244	8-act-DBA *		90	23.9	868.5569	868.5559	8-hode-DBA *	
17	5.9	942.4118	942.4084	8-gfr-BA *		91	23.9	882.5362	882.5317	8-lino-10-OH-BA	[8]
18	6.2	704.3277	704.3249	8-scn-BA	[2]	92	24.1	822.5151	822.5140	8-hpdde-BHA *	
19	6.3	674.3173	674.3172	8-scn-BHA *		93	24.2	834.5151	834.5136	8-linolen-BHA	[6]
20	6.6	732.3590	732.3601	8-adp-BA	[2]	94	24.2	844.5206	844.5218	8-pal-10-OH-BMA	[6]
21	6.7	702.3484	702.3473	8-adp-BHA *		95	24.3	806.5202	806.5190	8-lino-DMBHA *	
22	6.8	674.3535	674.3499	8-hbte-DBA *		96	24.3	784.4994	784.4973	8-myr-BHA	[19]
23	6.9	686.3171	686.3170	8-fmr-DBA *		97	24.4	820.5358	820.5348	8-lino-13-DMDBA *	
24	7.2	688.3691	688.3682	8-hvlr-DBA *		98	24.5	840.5256	840.5249	8-hpde-BMA	[8]
25	7.6	686.3171	686.3173	8-gtn-BHA *		99	24.6	814.5100	814.5087	8-ptdn-BMA	[6]
26	7.7	716.3641	716.3633	8-adp-DBA *		100	24.6	796.4994	796.4960	8-ptde-BHA	[8]
27	8.2	718.3433	718.3443	8-gtr-BA	[2]	101	24.7	854.5413	854.5778	8-hstr-DMA *	
28	8.7	658.3222	658.3244	8-bte-BMA *		102	24.8	852.5256	852.5254	8-lino-BMA	[6]
29	9.8	760.3903	760.3903	8-azl-BMA *		103	24.9	820.5358	820.5359	8-lino-3-DMDBA *	
30	10.3	744.4059	774.4051	8-sbc-BMA *		104	25.0	832.5358	832.5355	8-linolen-3,13-DDBA *	
31	10.8	644.3429	644.3423	8-btn-BHA *		105	25.0	802.5252	802.5237	8-linolen-DMDDBA *	
32	10.8	674.3535	674.3529	8-btn-BA *		106	25.1	836.5307	836.5293	8-lino-BHA	[6]
33	10.8	674.3535	674.3529	8-vlr-BMA*		107	25.1	810.5151	810.5145	8-pme-BHA	[8]
34	10.9	744.3954	744.3960	8-hnne-BMA *		108	25.3	866.5413	866.5408	8-lino-BA	[20]
35	11.4	902.5266	902.5268	8-thode-BMA *		109	25.3	870.5362	870.5372	8-ole-10-OH-BMA	[6]
36	11.5	758.4110	758.4119	8-azl-DBA *		110	25.5	808.5358	808.5306	8-ole-DMBHA *	
37	11.6	758.4110	758.4107	8-hnne-BA *		111	25.5	828.5256	828.5264	8-ptdn-BA	[6]
38	11.8	688.3691	688.3678	8-vlr-BA	[4]	112	25.7	858.5362	858.5352	8-pal-10-OH-BA	[6]
39	12.0	658.3586	658.3558	8-btn-DBA	[3]	113	25.9	798.5151	798.5128	8-ptdn-BHA	[6]
40	12.0	702.312	702.3845	8-hxn-BA	[4]	114	25.9	824.5307	824.5305	8-pme-DBA *	
41	12.0	658.3586	658.3599	8-vlr-BHA *		115	26.0	822.5515	822.5527	8-ole-13-DMDBA *	
42	12.2	916.5417	916.5399	8-thode-BA *		116	26.0	796.5358	796.5371	8-pal-DMDBA *	
43	12.3	886.5311	886.5662	8-thode-BHA *		117	26.1	822.5515	822.5511	8-ole-3-DMDBA *	
44	12.5	728.4004	728.3993	8-hnne-BHA *		118	26.1	828.5256	828.5251	8-pal-BMA	[20]
45	13.0	900.5468	900.5474	8-thode-DBA *		119	26.1	796.5358	796.5368	8-pal-13-DMDBA *	
46	13.2	790.4372	790.4367	8-dhudn-BMA		120	26.2	804.5409	804.5387	8-lino-DMDDBA *	
47	13.3	672.3742	672.3752	8-vlr-DBA *		121	26.2	884.5519	884.5494	8-ole-10-OH-BA	[6]
48	13.3	916.5417	916.5401	8-thnde-BMA *		122	26.3	854.5413	854.5403	8-ole-BMA	[6]
49	13.5	800.4216	800.4234	8-dded-BMA *		123	26.6	850.5464	850.5459	8-lino-DBA	[8]
50	13.6	774.4423	774.4400	8-dhudn-BHA		124	26.8	880.5569	880.5540	8-ecde-BMA	[12]
51	13.6	782.5202	782.5161	8-pal-DMBHA *		125	26.9	834.5515	834.5494	8-lino-3,13-DDBA	[18]
52	15.1	716.4004	716.3982	8-otn-BMA *		126	26.9	842.5413	842.5396	8-pal-BA	[20]
53	15.2	884.5155	884.5174	8-dhodd-BMA *		127	26.9	812.5307	812.5292	8-ptdn-DBA	[8]
54	15.9	730.4161	730.4154	8-nnn-BMA *		128	27.0	894.5362	894.5336	3-Acetyl-8-lino-BMA	[16]
55	16.2	898.5311	898.5307	8-dhodd-BA *		129	27.1	868.5569	868.5576	8-ole-BA	[20]
56	16.3	886.5311	886.5318	8-dhode-BMA*		130	27.2	838.5464	838.5443	8-ole-BHA	[6]
57	16.8	900.5468	900.5438	8-dhode-BA *		131	27.4	806.5565	806.5512	8-ole-DMDDBA *	
58	17.0	866.5049	866.5054	8-hodt-BMA *		132	27.5	812.5307	812.5300	8-pal-BHA	[6]
59	17.1	868.5206	868.5191	8-dhodd-BHA *		133	27.6	856.5569	856.5553	8-str-BMA	[10]
60	17.3	888.5468	888.5438	8-dhstr-BMA *		134	27.7	836.5619	836.5712	8-ole-3,13-DDBA *	
61	17.4	870.5362	870.5335	8-dhode-BHA *		135	28.0	826.5464	826.5465	8-pal-DBA	[6]
62	18.1	884.5313	884.5292	8-hodd-10-OH-BMA *		136	28.2	926.6352	926.6308	8-tcn-BMA *	
63	18.1	884.5519	884.5486	8-dhode-DBA *		137	28.3	780.5409	780.5384	8-pal-DMDDBA *	
64	18.4	880.5206	880.5199	8-hodt-BA *		138	28.4	842.5413	842.5399	8-hpdn-BMA	[6]
65	18.4	850.500	850.5096	8-hodt-BHA *		139	28.5	852.5620	852.5620	8-ole-DBA	[6]
66	18.5	872.5519	872.5518	8-dhstr-BHA *		140	28.9	912.6195	912.6194	8-dcn-BMA *	
67	18.8	868.5206	868.5196	8-hodd-BMA *		141	29.0	810.5515	810.5490	8-pal-3,13-DDBA	[8]
68	18.8	910.5311	910.5304	8-dhecte-BMA *		142	29.7	870.5726	870.5569	8-str-BA	[20]
69	19.4	882.5368	882.5349	8-hodd-BA *		143	29.7	954.6665	954.6648	8-ttcn-BA	[5]
70	19.8	924.5548	924.5455	8-dhhctte-BA *		144	30.5	840.5620	840.5583	8-hpdn-DBA	[8]
71	20.1	852.5256	852.5264	8-hodd-BHA *		145	30.5	840.5620	840.5636	8-str-BHA	[8]
72	20.1	870.5362	870.5359	8-hode-BMA *		146	30.5	940.6508	940.6506	8-ttcn-BMA	[21]
73	21.0	864.5256	864.5255	8-hodt-DBA *		147	30.9	926.6279	926.6308	8-dcn-BA	[8]
74	21.2	866.5413	866.5410	8-hodd-DBA *		148	31.6	896.6246	896.6225	8-dcn-BHA	[21]

Abbreviations: fmr (fumaric acid), bte (butenoic acid), hbte (hydroxybutenoic acid), scn (succinic acid), btn (butanoic acid), hbtn (hydroxybutanoic acid), dhbtn (dihydroxybutanoic acid), gtn (glutaconic acid), ogtr (oxoglutaric acid), gtr (glutaric acid), vlr (valeric acid), hvlr (hydroxyvaleric acid), act (aconitic acid), adp (adipic acid), hadp (hydroxyadipic acid), hxn (hexanoic acid), dohpnd (dioxoheptanedioic acid), otn (octanoic acid), hnne (hydroxynonenoic acid), azl (azelaic acid), nnn (nonanoic acid), sbc (sebacic acid), dhudn (dihydroxyundecanoic acid), dded (dodecenedioic acid), laur (lauric acid), myr (myristic acid), ptde (pentadecenoic acid), ptdn (pentadecanoic acid), pmde (palmitadienoic acid), pme (palmitoleic acid), pal (palmitic acid), hpdde (heptadecadienoic acid), hpde (heptadecenoic acid), hpdn (heptadecanoic acid), linolen (linolenic acid), hodt (hydroxyoctadecatrienoic acid), lino (linoleic acid), hodd (hydroxyoctadecadienoic acid), dhodd (dihydroxyloctadecadienoic acid), ole (oleic acid), hode (hydroxyoctadecenoic acid), dhode (dihydroxyoctadecenoic acid), thode (trihydroxyoctadecenoic acid), str (stearic acid), hstr (hydroxystearic acid), dhstr (dihydroxy stearic acid), thnde (trihydroxynonadecenoic acid), dhecte (dihydroxyeicosatrienoic acid), ecde (eicosadienoic acid), dhhctte (dihydroxyhenicosatetraenoic acid), dcn (docosanoic acid), tcn (tricosanoic acid), ttcn (tetracosanoic acid), gvl (glucovanillic acid), gcf (glucocaffeic acid), gfr (glucoferulic acid). * New compounds.

**Table 3:** Fatty acid side chains in lipo-alkaloids.

No.	MF	Possible Structure	No. of LAs	Ref.	No.	MF	Possible Structure	No. of LAs	Ref.
1	C_3_H_6_O_2_	Propanoic acid	ND	[1]	40	C_17_H_30_O_2_ *	Heptadecadienoic acid	2	
2	C_4_H_4_O_4_	Fumaric acid	2	[11]	41	C_17_H_32_O_2_	Heptadecenoic acid	1	[1]
3	C_4_H_6_O_2_ *	Butenoic acid	1		42	C_17_H_34_O_2_	Heptadecanoic acid	2	[1]
4	C_4_H_6_O_3_ *	Hydroxybutenoic acid	1		43	C_18_H_30_O_2_	Linolenic acid	9	[1]
5	C_4_H_6_O_4_	Succinic acid	2	[1,2]	44	C_18_H_30_O_3_ *	Hydroxyoctadecatrienoic acid	4	
6	C_4_H_6_O_5_	Malic acid	ND		45	C_18_H_32_O_2_	Linoleic acid	12	[1]
7	C_4_H_8_O_2_	Butanoic acid	3	[11]	46	C_18_H_32_O_3_ *	Hydroxyoctadecadienoic acid	5	
8	C_4_H_8_O_3_	Hydroxybutanoic acid	3	[11]	47	C_18_H_32_O_4_ *	Dihydroxyoctadecadienoic acid	3	
9	C_4_H_8_O_4_ *	Dihydroxybutanoic acid	1		48	C_18_H_34_O_2_	Oleic acid	11	[1]
10	C_5_H_6_O_4_	Glutaconic acid	2	[11]	49	C_18_H_34_O_3_ *	Hydroxyoctadecenoic acid	4	
11	C_5_H_6_O_5_ *	Oxoglutaric acid	1		50	C_18_H_34_O_4_ *	Dihydroxyoctadecenoic acid	4	
12	C_5_H_8_O_4_	Glutaric acid	1	[2]	51	C_18_H_34_O_5_ *	Trihydroxyoctadecenoic acid	4	
13	C_5_H_10_O_2_	Valeric acid	4		52	C_18_H_36_O_2_	Stearic acid	3	[1]
14	C_5_H_10_O_3_ *	Hydroxyvaleric acid	1		53	C_18_H_36_O_3_ *	Hydroxystearic acid	1	
15	C_6_H_6_O_6_ *	Aconitic acid	5		54	C_18_H_36_O_4_ *	Dihydroxy stearic acid	2	
16	C_6_H_10_O_4_	Adipic acid	3	[2,11]	55	C_19_H_32_O_2_	Nonadecatrienoic acid	ND	[8]
17	C_6_H_10_O_5_ *	Hydroxyadipic acid	5		56	C_19_H_34_O_2_	Nonadecadienoic acid	ND	[1]
18	C_6_H_12_O_2_	Hexanoic acid	1		57	C_19_H_36_O_2_	Nonadecenoic acid	ND	[1]
19	C_7_H_8_O_4_ *	Heptadienedioic acid	1		58	C_19_H_36_O_5_ *	Trihydroxynonadecenoic acid	1	
20	C_7_H_8_O_6_ *	Dioxoheptanedioic acid	1		59	C_19_H_38_O_2_	Nonadecanoic acid	ND	[1]
21	C_7_H_12_O_4_	Pimelic acid	ND	[2]	60	C_20_H_30_O_2_	Eicosapentaenoic acid	ND	[1]
22	C_8_H_14_O_4_	Suberic acid	ND	[2]	61	C_20_H_32_O_2_	Eicosatetraenoic acid	ND	[1]
23	C_8_H_16_O_2_ *	Octanoic acid	1		62	C_20_H_34_O_2_	Eicosatrienoic acid	ND	[1]
24	C_9_H_16_O_3_ *	Hydroxynonenoic acid	3		63	C_20_H_34_O_4_ *	Dihydroxyeicosatrienoic acid	1	
25	C_9_H_16_O_4_	Azelaic acid	2	[12]	64	C_20_H_36_O_2_	Eicosadienoic acid	1	[1]
26	C_9_H_18_O_2_ *	Nonanoic acid	1		65	C_20_H_38_O_2_	Eicosenoic acid	ND	[1]
27	C_10_H_18_O_4_	Sebacic acid	1	[2]	66	C_20_H_40_O_2_	Eicosanoic acid	ND	[1]
28	C_11_H_20_O_4_	Undecanedioic acid	ND	[2]	67	C_21_H_32_O_2_	Henicosapentaenoic acid	ND	[1]
29	C_11_H_22_O_4_ *	Dihydroxyundecanoic acid	2		68	C_21_H_34_O_2_	Henicosatetraenoic acid	ND	[1]
30	C_12_H_20_O_4_ *	Dodecenedioic acid	1		69	C_21_H_34_O_4_ *	Dihydroxyhenicosatetrenoic acid	1	
31	C_12_H_24_O_2_	Lauric acid	1	[1]	70	C_22_H_32_O_2_	Docosahexaenoic acid	ND	[1]
32	C_14_H_24_O_2_	Tetradecadienoic acid	ND	[1]	71	C_22_H_44_O_2_	Docosanoic acid	3	[1]
33	C_14_H_26_O_2_	Tetradecenoic acid	ND	[1]	72	C_23_H_42_O_2_	Tricosadienoic Acid	ND	[1]
34	C_14_H_28_O_2_	Myristic acid	1	[1]	73	C_23_H_44_O_2_	Tricosenoic Acid	ND	[1]
35	C_15_H_28_O_2_	Pentadecenoic acid	2	[1]	74	C_23_H_46_O_2_ *	Tricosanoic Acid	1	
36	C_15_H_30_O_2_	Pentadecanoic acid	4	[1]	75	C_24_H_46_O_2_	Tetracosenoic acid	ND	[1]
37	C_16_H_28_O_2_	Palmitadienoic acid	1	[1]	76	C_24_H_48_O_2_	Tetrecosanoic acid	2	[1]
38	C_16_H_30_O_2_	Palmitoleic acid	4	[1]	77	C_25_H_50_O_2_	Pentacosanoic acid	ND	[1]
39	C_16_H_32_O_2_	Palmitic acid	11	[1]					

* Indicates the side chains detected for the first time; MF: molecular formula; LAs: lipo-alkaloids; ND: not detected.

## Supplementary Materials

Supplementary materials can be accessed at: http://www.mdpi.com/1420-3049/21/4/437/s1.

## Abbreviation

The following abbreviations are used in this manuscript:

UHPLC-Q-TOF-MS	ultra-high performance liquid chromatography-quadrupole-time of flight-mass spectrometry
BA	14-benzoylaconine
BMA	14-benzoylmesaconine
BHA	14-benzoylhypaconine
10-OH-BA	10-hydroxy-14-benzoylaconine
3-DBA	3-deoxy-14-benzoylaconine
15-DBA	15-deoxy-14-benzoylaconine
3,13-DDBA	3,13-dideoxy-14-benzoylaconine
10-OH-BMA	10-hydroxy-14-benzoylmesaconine
15-DBHA	15-deoxy-14-benzoylhypaconine
3-Ac-10-OH-BA	3-acetyl-10-hydroxy-14-benzoylaconine
3-Ac-10-OH-BMA	3-acetyl-10-hydroxy-14-benzoylmesaconine
3-Ac-BA	3-acetyl-14-benzoylaconine
3-Ac-BMA	3-acetyl-14-benzoylmesaconine
3-DMDBA	demethoxy-3-deoxy-14-benzoylaconine
13-DMDBA	demethoxy-13-deoxy-14-benzoylaconine
DMDDBA	demethoxy-3,13-dideoxy-14-benzoylaconine
DMBHA	demethoxy-14-benzoylhypaconine
