# Supplementary material for: Identification of Oxygenated Fatty Acid as a Side Chain of Lipo-Alkaloids in Aconitum carmichaelii by UHPLC-Q-TOF-MS and a Database
Source: Molecules. 2016 Mar 31;21(4):437. doi: 10.3390/molecules21040437 (PMC6273321; doi:10.3390/molecules21040437)
Supplement: Supplementary file 1 [file molecules-21-00437-s001.pdf]

Supplementary Materials: Identification of Oxygenated Fatty Acid as a Side Chain of Lipo-Alkaloids in *Aconitum carmichaelii* by UHPLC-Q-TOF-MS and a Database

Ying Liang, Jian-Lin Wu, Elaine Lai-Han Leung, Hua Zhou, Zhongqiu Liu, Guanyu Yan, Ying Liu, Liang Liu and Na Li

Table S1. ^{1}H-NMR and ^{13}C-NMR spectroscopic data for Compounds **A1–A6** (δ in ppm and *J* in Hz).

Position	A1		A2	A3	A4	A5	A6
	δ_H (ppm)	δ_C (ppm)	δ_H (ppm)	δ_H (ppm)	δ_H (ppm)	δ_H (ppm)	δ_H (ppm)
1	3.141 (1H, dd, J_1 = 8.4, J_2 = 6)	83.47	3.163 (1H, m)	3.148 (1H, t, J = 9.0)	3.154 (1H, m)	3.173 (1H, m)	3.156 (1H, m)
2	2.151 (1H, m), 2.327 (1H, J = 9.6)	34.18	1.460 (1H, m), 1.810 (1H, m)	2.155 (1H, m), 2.332 (1H, d, J = 11.4)	2.161 (1H, m), 2.337 (1H, m)	2.386 (1H, m)	2.382 (1H, m)
3	3.790 (1H, m)	71.37	3.824 (1H, m)	3.795 (1H, m)	3.788 (1H, m)	3.824 (1H, m)	3.807 (1H, dd, J_1 = 9.0, J_2 = 4.8)
4	-	43.64	-	-	-	-	-
5	2.094 (1H, d, J = 6.6)	46.52	2.157 (1H, br s)	2.103 (1H, d, J = 6.6)	2.099 (1H, d, J = 6)	2.161 (1H, br s)	2.152 (1H, br s)
6	4.049 (1H, d, J = 6.6)	82.62	4.051 (1H, d, J = 6.6)	4.050 (1H, d, J = 6.6)	4.051 (2H, d, J = 6.6)	4.055 (1H, d, J = 6.6)	4.049 (1H, d, J = 6.0)
7	2.903 (1H, s)	44.54	2.862 (1H, s)	2.906 (1H, s)	2.907 (1H, s)	2.865 (1H, s)	2.869 (1H, s)
8	-	91.71	-	-	-	-	-
9	2.936 (1H, t, J = 5.4)	43.93	2.929 (1H, t, J = 5.4)	2.936 (1H, t, J = 5.4)	2.939 (1H, t, J = 5.4)	2.931 (1H, t, J = 6.0)	2.934 (1H, t, J = 5.4)
10	2.138 (1H, m)	41.11	2.168 (1H, m)	2.144 (1H, d, J = 3.0)	2.146 (1H, d, J = 4.2)	2.173 (1H, m)	2.168 (1H, m)
11	-	50.25	-	-	-	-	-
12	2.325 (1H, m), 2.828 (1H, m)	34.93	/	/	/	/	/
13	-	74.22	-	-	-	-	-
14	4.876 (1H, d, J = 4.8)	79.14	4.882 (1H, d, J = 5.4)	4.885 (1H, d, J = 4.8)	4.878 (1H, d, J = 5.4)	4.885 (1H, d, J = 4.8)	4.882 (1H, d, J = 4.9)
15	4.452 (1H, dd, J_1 = 5.4, J_2 = 3.0)	79.09	4.461 (1H, dd, J_1 = 5.4, J_2 = 2.4)	4.455 (1H, dd, J_1 = 5.4, J_2 = 2.4)	4.454 (1H, dd, J_1 = 5.4, J_2 = 2.4)	4.464 (1H, dd, J_1 = 5.4, J_2 = 2.4)	4.461 (1H, dd, J_1 =5.4, J_2 = 3.0)
15-OH	4.475 (1H, d, J = 2.4)		4.517 (1H, d, J = 2.6)	4.522 (1H, d, J = 2.4)	4.475 (1H, d, J = 2.4)	4.522 (1H, d, J = 2.0)	4.518 (1H, d, J = 2.6)
16	3.352 (1H, d, J = 5.4)	90.27	3.364 (1H, d, J = 5.4)	3.354 (1H, d, J = 4.8)	3.353 (1H, d, J = 5.4)	3.365 (1H, d, J = 5.4)	3.372 (1H, d, J = 4.8)
17	3.056 (1H, s)	62.38	3.117 (1H, s)	3.061 (1H, s)	3.063 (1H, s)	3.109 (1H, s)	3.116 (1H, s)
18	3.553 (1H, d, J = 9.0) 3.641 (1H, d, J = 9.0)	76.45	3.481 (1H, d, J = 9.0) 3.621 (1H, d, J = 9.0)	3.552 (1H, d, J = 9.0) 3.643 (1H, d, J = 9.0)	3.554 (1H, d, J = 9.0) 3.644 (1H, d, J = 9.0)	3.485 (1H, d, J = 9.0 3.623 (1H, d, J = 9.0)	3.484 (1H, d, J = 8.9) 3.623 (1H, d, J = 8.9)
19	3.142 (1H, dd, J_1 = 8.4, J_2 = 6), 3.786 (1H, dd, J_1 = 12.0, J_2 = 7.2)	49.52	-	-	-	-	-
N-CH_3	2.334 (3H, s)	42.57	-	2.379 (3H, s)	2.374 (3H, s)	-	-
N-$CH_2\underline{CH_3}$	-	-	1.125 (3H, t, J = 7.2)	-	-	1.131 (3H, t, J = 6.6)	1.116 (3H, t, J = 7.2)
1-OMe	3.356 (3H, s)	56.46	3.320 (3H, s)	3.319 (3H, s)	3.321 (3H, s)	3.340 (3H, s)	3.321 (3H, s)
6-OMe	3.172 (3H, s)	58.28	3.177 (3H, s)	3.175 (3H, s)	3.175 (3H, s)	3.181 (3H, s)	3.177 (3H, s)
16-OMe	3.782 (3H, s)	61.33	3.785 (3H, s)	3.771 (3H, s)	3.773 (3H, s)	3.787 (3H, s)	3.796 (3H, s)
18-OMe	3.306 (3H, s)	59.25	3.287 (3H, s)	3.309 (3H, s)	3.310 (3H, s)	3.291 (3H, s)	3.285 (3H, s)
14-O-C=O	-	166.18	-	-	-	-	-

Table S1. *Cont.*

Position	A1		A2	A3	A4	A5	A6
	δ_H (ppm)	δ_C (ppm)	δ_H (ppm)	δ_H (ppm)	δ_H (ppm)	δ_H (ppm)	δ_H (ppm)
1′	-	129.87	-	-	-	-	-
2′,6′	8.049 (2H, d, *J* = 7.2)	129.87	8.047 (2H, t, *J* = 7.4)	8.048 (2H, d, *J* = 7.6)	8.052 (2H, d, *J* = 7.2)	8.051 (1H, d, *J* = 7.2)	8.051 (2H, d, *J* = 7.2)
3′,5′	7.471 (2H, t, *J* = 7.8)	128.78	7.471 (2H, t, *J* = 7.4)	7.472 (2H, t, *J* = 7.6)	7.473 (2H, t, *J* = 7.6)	7.475 (1H, t, *J* = 7.2)	7.472 (2H, t, *J* = 7.6)
4′	7.589 (1H, t, *J* = 7.4)	133.41	7.590 (1H, t, *J* = 7.4)	7.591 (1H, t, *J* = 7.2)	7.592 (1H, t, *J* = 7.2)	7.593 (1H, t, *J* = 7.2)	7.591 (1H, t, *J* = 7.2)
(8-)-1″	-	175.3	-	-	-	-	-
2″	1.450 (1H, m), 1.816 (1H, m)	34.92	1.473 (1H, m), 1.817 (1H, m)	1.456 (1H, m), 1.819 (1H, m)	1.451 (1H, m), 1.818 (1H, m)	1.483 (1H, m), 1.820 (1H, m)	1.487 (1H, m), 1.817 (1H, m)
3″	1.025 (1H, m), 1.179 (1H, m)	24.29	1.025 (1H, m), 1.174 (1H, m)	1.015 (1H, m), 1.152 (1H, m)	1.020 (1H, m), 1.173 (1H, m)	1.012 (1H, m), 1.204 (1H, m)	1.010 (1H, m), 1.172 (1H, m)
4″	0.885 (2H, m)	29.16	0.887 (2H, m)	0.873 (2H, m)	0.884 (2H, m)	0.888 (2H, m)	0.814 (2H, m)
5″	1.026 (2H, m)	29.12	1.025 (2H, m)	1.011 (2H, m)	1.023 (2H, m)	1.012 (2H, m)	1. 012(2H, m)
6″	1.177 (2H, m)	29.48	1.174 (2H, m)	1.144 (2H, m)	1.176 (2H, m)	1.204 (2H, m)	1.173(2H, m)
7″	1.380 (2H, m)	29.73	1.324 (2H, m)	1.229 (2H, m)	1.312 (2H, m)	1.311 (2H, m)	1.306 (2H, m)
8″	2.045 (2H, m)	27.31	2.058 (2H, m)	1.229 (2H, m)	2.042 (2H, m)	1.311 (2H, m)	2.034 (2H, m)
9″	5.376 (1H, m)	130.23	5.380 (1H, m)	1.229 (2H, m)	5.374 (1H, m)	1.311 (2H, m)	5.384 (1H, m)
10″	5.376 (1H, m)	128.29	5.380 (1H, m)	1.229 (2H, m)	5.374 (1H, m)	1.311 (2H, m)	5.384 (1H, m)
11″	2.802 (2H, m)	25.80	2.797 (2H, t, *J* = 6.0)	1.229 (2H, m)	2.042 (2H, m)	1.311 (2H, m)	2.034 (2H, m)
12″	5.376 (1H, m)	128.03	5.380 (1H, m)	1.229 (2H, m)	1.312 (2H, m)	1.311 (2H, m)	1.306 (2H, m)
13″	5.376 (1H, m)	130.44	5.380 (1H, m)	1.229 (2H, m)	1.312 (2H, m)	1.311 (2H, m)	1.306 (2H, m)
14″	2.045 (2H, m)	27.37	2.058 (2H, m)	1.329 (2H, m)	1.312 (2H, m)	1.311 (2H, m)	1.306 (2H, m)
15″	1.380 (2H, m)	29.00	1.324 (2H, m)	1.229 (2H,m)	1.312 (2H, m)	1.311 (2H, m)	1.306 (2H, m)
16″	1.380 (2H, m)	31.68	1.324 (2H, m)	0.907 (3H, t, *J* = 6.6)	1.312 (2H, m)	0.915 (3H, t, *J* = 7.2)	1.306 (2H, m)
17″	1.308 (2H, m)	22.71	1.324 (2H, m)	-	1.312 (2H, m)	-	1.306 (2H, m)
18″	0.907 (3H, t, *J* = 6.6)	14.20	0.908 (3H, t, *J* = 7.2)	-	0.906 (3H, t, *J* = 6.6)	-	0.910 (3H, t, *J* = 7.2)

Table S2. Retention times, MS and MS/MS fragmentation ions for the identified lipo-alkaloids in *A. carmichaelii*.

No.	Alkaloid	Rt (min)	Calculated [M + H]+	Observed [M + H]+	MS/MS Fragmentation Ions
1	8-dhbtn-BMA *	3.4	692.3277	692.3285	572.2842, 540.2570, 522.2476, 512.2633, 508.2308, 490.2205, 354.1695, 105.0340
2	8-act-10-OH-BMA *	3.4	762.2968	762.2968	588.2792, 556.2521, 538.2408, 528.2580, 524.2264, 506.2159, 496.2305, 492.1995, 370.1640, 105.0342
3	8-gvl-BMA *	3.6	902.3805	902.3777	572.2847, 540.2575, 522.2483, 512.2639, 508.2298, 490.2220, 480.2344, 354.1707, 105.0338
4	8-act-BMA *	3.8	746.3018	746.3002	572.2842, 540.2582, 522.2462, 512.2626, 508.2344, 490.2192, 480.2367, 476.2047, 354.1695, 105.0335
5	8-hbtn-10-OH-BMA *	4.0	692.3277	692.3258	588.2798, 556.2524, 538.2397, 528.2573, 524.2261, 506.2156, 496.2288, 370.1646, 105.0329
6	8-gcf-BMA *	4.2	914.3805	914.3784	752.3262, 572.2838, 540.2586, 522.2462, 512.2634, 508.2324, 490.2216, 476.2046, 354.1702, 105.0335
7	8-gfr-BMA *	4.5	928.3961	928.3946	766.3412, 572.2842, 540.2574, 522.2469, 512.2642, 508.2329, 490.2207, 480.2325, 476.2111, 354.1687, 105.0336
8	8-act-BHA *	4.6	730.3069	730.3041	556.2928, 524.2662, 496.2698, 492.2375, 464.2452, 460.2083, 338.1737, 105.0331
9	8-adp-10-OH-BMA *	4.7	734.3382	734.3324	588.2802, 556.2539, 538.2799, 528.2597, 524.2285, 506.2531, 496.2327, 492.2020, 370.1651, 105.0336
10	8-hadp-BMA *	4.7	734.3382	734.3324	572.2848, 540.2605, 522.2419, 512.2612, 508.2277, 354.1695, 105.0345
11	8-hbtn-BMA *	4.8	676.3328	676.3321	572.2845, 540.2590, 522.2472, 512.2631, 508.2319, 490.2212, 480.2368, 354.1699, 105.0335
12	8-act-BA *	5.0	760.3175	760.3189	586.3000, 554.2730, 536.2644, 526.2794, 522.2483, 504.2426, 494.2519, 490.2192, 368.1853, 105.0337
13	8-dohpnd-BMA *	5.3	760.3175	760.3176	572.2837, 540.2591, 522.2483, 512.2639, 508.2324, 490.2192, 480.2364, 476.217, 354.1699, 105.0337
14	8-fmr-BHA *	5.6	672.3015	672.3024	556.2890, 524.2636, 496.2685, 492.2386, 464.2441, 460.2147, 338.1754, 105.0335
15	8-hbtn-BHA *	5.8	660.3384	660.3380	556.296, 524.2632, 496.2673, 492.2372, 464.2429, 460.2101, 338.1748, 105.0341
16	8-act-DBA *	5.8	744.3226	744.3244	570.3048, 538.2789, 510.2838, 506.2522, 478.2573, 352.1903, 105.0336
17	8-gfr-BA *	5.9	942.4118	942.4084	780.3579, 586.2997, 554.2729, 536.2591, 526.2775, 522.2464, 504.2341, 494.2538, 368.1844, 105.0335
19	8-scn-BHA *	6.3	674.3173	674.3172	556.2894, 524.2633, 496.2681, 492.2369, 464.2421, 460.2110, 338.1745, 105.0342
21	8-adp-BHA *	6.7	702.3484	702.3473	556.2885, 524.2621, 496.2662, 492.2359, 464.2415, 460.2072, 338.1744, 105.0339
22	8-hbte-DBA *	6.8	674.3535	674.3499	570.3052, 538.2791, 522.2800, 510.2842, 508.2592, 352.1911, 105.0341
23	8-fmr-DBA *	6.9	686.3171	686.3170	570.3050, 538.2779, 512.2855, 508.2626, 352.1906, 105.0334
24	8-hvlr-DBA *	7.2	688.3691	688.3682	570.3057, 538.2797, 522.2470, 512.2624, 508.2319, 490.2226, 352.1905, 105.0335
25	8-gtn-BHA *	7.6	686.3171	686.3173	556.2896, 524.2625, 496.2762, 492.2368, 464.2416, 460.2141, 338.1745, 105.0336
26	8-adp-DBA *	7.7	716.3641	716.3633	570.3047, 538.2812, 522.2548, 512.2552, 508.2592, 354.1648, 105.0342
28	8-bte-BMA *	8.7	658.3222	658.3244	572.2848, 540.2599, 522.2465, 512.2658, 508.2337, 490.2222, 480.2378, 354.1695, 105.0338
29	8-azl-BMA *	9.8	760.3903	760.3903	572.2833, 540.2566, 522.2426, 512.2661, 508.2354, 354.1685, 105.0339
30	8-sbc-BMA *	10.3	744.4059	774.4051	572.2850, 540.2585, 522.2487, 512.2639, 508.2336, 490.2213, 480.2391, 476.2074, 354.1691, 105.0332
31	8-btn-BHA *	10.8	644.3429	644.3423	556.2900, 524.2634, 496.2678, 492.2367, 464.2414, 460.2104, 338.1748, 105.0339
32	8-btn-BA *	10.8	674.3535	674.3529	586.3011, 554.2742, 536.2633, 526.2794, 522.2848, 512.2645, 508.2336, 490.2217, 368.1853, 105.0338
33	8-vlr-BMA *	10.8	674.3535	674.3529	572.2845, 540.2579, 522.2848, 512.2645, 508.2336, 490.22117, 480.2374, 354.1697, 105.0338
34	8-hnne-BMA *	10.9	744.3954	744.3960	572.2841, 540.2590, 522.2478, 512.2631, 490.2193, 480.2344, 354.1687, 105.0343
35	8-thode-BMA *	11.4	902.5266	902.5268	572.2844, 540.2580, 522.2470, 512.2626, 508.2316, 490.2202, 480.2352, 354.1688, 105.0335
36	8-azl-DBA *	11.5	758.4110	758.4119	570.3059, 538.2772, 510.2757, 506.2489, 478.2550, 352.1893, 105.0335
37	8-hnne-BA *	11.6	758.4110	758.4103	586.3000, 554.2769, 536.2622, 526.2731, 522.2481, 494.2670, 490.2187, 368.1851, 105.0335
39	8-btn-DBA	12.0	658.3586	658.3558	570.3050, 538.2789, 522.2867, 512.2920, 508.2709, 490.2562, 352.1905, 105.0334

Table S2. *Cont.*

No.	Alkaloid	Rt (min)	Calculated [M + H]+	Observed [M + H]+	MS/MS Fragmentation Ions
41	8-vlr-BHA *	12.0	658.3586	658.3599	556.2898, 524.2636, 496.2677, 492.2373, 464.2420, 460.2112, 338.1749, 105.0334
42	8-thode-BA *	12.2	916.5417	916.5399	586.2996, 554.2718, 536.2638, 526.2778, 368.1835, 105.0335
43	8-thode-BHA *	12.3	886.5311	886.5662	556.2882, 524.2624, 496.2666, 492.2363, 464.2410, 460.2115, 338.1736, 105.0335
44	8-hnne-BHA *	12.5	728.4004	728.3993	556.2896, 524.2635, 496.2667, 492.2362, 464.2414, 460.2092, 338.1752, 105.0336
45	8-thode-DBA *	13.0	900.5468	900.5474	570.3066, 538.2801, 510.2856, 506.2533, 478.2591, 352.1910, 105.0336
46	8-dhudn-BMA	13.2	790.4372	790.4367	572.2851, 540.2601, 522.2500, 512.2661, 508.2323, 354.1705, 105.0336
47	8-vlr-DBA *	13.3	672.3742	672.3752	570.3055, 538.2791, 522.2834, 512.2871, 508.2557, 490.2582, 352.1903, 105.0332
48	8-thnde-BMA *	13.3	916.5417	916.5401	572.2851, 540.2596, 522.2480, 512.2632, 508.2313, 490.2171, 480.2359, 354.1699, 105.0334
49	8-dded-BMA *	13.5	800.4216	800.4234	572.2828, 540.2594, 522.2456, 512.2590, 508.2293, 354.1679, 105.0339
50	8-dhudn-BHA	13.6	774.4423	774.4400	556.2895, 524.2638, 496.2680, 492.2361, 464.2439, 338.1739, 105.0336
51	8-pal-DMBHA *	13.6	782.5202	782.5161	526.2761, 494.2505, 462.2232, 434.2322, 340.1876, 105.0330
52	8-otn-BMA *	15.1	716.4004	716.3982	572.2849, 540.2582, 522.2449, 512.2623, 508.2323, 490.2213, 480.2370, 476.2048, 354.1691, 105.0336
53	8-dhodd-BMA *	15.2	884.5155	884.5174	572.2851, 540.2615, 522.2497, 512.2631, 508.2316, 490.2222, 480.2374, 354.1707, 105.0335
54	8-nnn-BMA *	15.9	730.4161	730.4154	572.2837, 540.2580, 522.2492, 512.2614, 508.2317, 490.2202, 480.2357, 476.2021, 354.1688, 105.0329
55	8-dhodd-BA *	16.2	898.5311	898.5307	586.3003, 554.2745, 536.2634, 526.2801. 522.2473, 504.2414, 494.2503, 368.1860, 105.0334
56	8-dhode-BMA *	16.3	886.5311	886.5318	572.2847, 540.2583, 522.2474, 512.2629, 508.2323, 490.2208, 480.2368, 354.1694, 105.0334
57	8-dhode-BA *	16.8	900.5468	900.5438	586.3003, 554.2741, 536.2634, 526.2789, 522.2481, 504.2376, 494.2521, 368.1859, 105.0335
58	8-hodt-BMA *	17.0	866.5049	866.5054	572.2847, 540.2578, 522.2469, 512.2628, 508.2329, 490.2214, 480.2367, 354.1693, 105.0335
59	8-dhodd-BHA *	17.1	868.5206	868.5191	556.2904, 524.2636, 496.2678, 492.2375, 464.2415, 460.2123, 338.1749, 105.0335
60	8-dhstr-BMA *	17.3	888.5468	888.5438	572.2850, 540.2584, 522.2483, 512.2637, 508.2325, 490.2210, 480.2358, 354.1699, 105.0335
61	8-dhode-BHA *	17.4	870.5362	870.5335	556.2904, 524.2634, 496.2681, 492.2373, 464.2414, 460.2097, 338.1749, 105.0335
62	8-hodd-10-OH-BMA *	18.1	884.5313	884.5292	588.2794, 556.2874, 538.2781, 528.2580, 524.2602, 506.2514, 370.1641, 105.0334
63	8-dhode-DBA *	18.1	884.5519	884.5486	570.3050, 538.2781, 510.2830, 506.2514, 478.2579, 352.1905, 105.0336
64	8-hodt-BA *	18.4	880.5206	880.5199	586.3009, 554.2742, 536.2633, 526.2796, 522.2457, 504.2389, 494.2531, 368.1858, 105.0336
65	8-hodt-BHA *	18.4	850.5100	850.5096	556.2904, 524.2638, 496.2686, 492.2372, 464.2414, 460.2108, 338.1747, 105.0335
66	8-dhstr-BHA *	18.5	872.5519	872.5518	556.2903, 524.2642, 496.2694, 492.2375, 464.2429, 460.2103, 338.1751, 105.0335
67	8-hodd-BMA *	18.8	868.5206	868.5196	572.2850, 540.2584, 522.2472, 512.2635, 508.2315, 490.2199, 480.2384, 354.1692, 105.0336
68	8-dhecte-BMA *	18.8	910.5311	910.5304	572.2849, 540.2595, 522.2465, 512.2636, 508.2310, 490.2198, 480.2382, 354.1699, 105.0338
69	8-hodd-BA *	19.4	882.5368	882.5349	586.3005, 554.2735, 536.2622, 526.2782, 522.2465, 504.2361, 494.2516, 368.1847, 105.0336
70	8-dhhctte-BA *	19.8	924.5548	924.5455	556.2896, 524.2627, 496.2682, 492.2367, 464.2418, 460.2135, 338.1746, 105.0342
71	8-hodd-BHA *	20.1	852.5256	852.5264	556.2907, 524.2642, 496.2681, 492.2371, 464.2422, 460.2104, 338.1747, 105.0335
72	8-hode-BMA *	20.1	870.5362	870.5359	572.2849, 540.2585, 522.2472, 512.2628, 508.2319, 490.2213, 480.2387, 354.1693, 105.0336
73	8-hodt-DBA *	21.0	864.5256	864.5255	570.3047, 538.2783, 510.2831, 506.2516, 478.2566, 352.1897, 105.0334
74	8-hodd-DBA *	21.2	866.5413	866.541	570.3047, 538.2797, 510.2843, 506.2522, 478.2584, 352.1906, 105.0335
75	8-hode-BA *	21.8	884.5524	884.5494	586.3002, 554.2727, 536.2628, 526.2781,522.2470, 504.2360, 494.2516, 368.1843, 105.0336
79	8-hode-BHA *	22.7	854.5413	854.5409	556.2903, 524.2638, 496.2679, 492.2371, 464.2424, 460.2113, 338.1747, 105.0334
85	8-linolen-3-DMDBA *	23.3	818.5202	818.5209	540.2948, 508.2693, 480.2710, 476.2424, 448.2476, 354.3065, 105.0330
88	8-hpdde-BMA *	23.8	838.5100	838.5105	572.2847, 540.2607, 522.2475, 512.2635, 508.2329, 490.2216, 480.2362, 354.1696, 105.0333
90	8-hode-DBA *	23.9	868.5569	868.5559	570.3029, 538.2776, 510.2792, 352.1858, 105.0336
92	8-hpdde-BHA *	24.1	822.5151	822.514	556.2901, 524.2640, 496.2685, 492.2396, 464.2423, 460.2320, 338.1756, 105.0337
95	8-lino-DMBHA *	24.3	806.5202	806.519	526.2758, 494.2499, 462.2244, 434.2319, 340.1882, 105.0332
97	8-lino-13-DMDBA *	24.4	820.5358	820.5348	540.2966, 508.2707, 490.2588, 480.2750, 476.2435, 458.2327, 448.2482, 105.0337

Table S2. *Cont.*

No.	Alkaloid	Rt (min)	Calculated [M + H]+	Observed [M + H]+	MS/MS Fragmentation Ions
101	8-hstr-DMA *	24.7	854.5726	854.5778	570.3042, 538.2789, 510.2845, 506.2506, 478.2553, 352.1900, 105.0333
103	8-lino-3-DMDBA *	24.9	820.5358	820.5359	540.2960, 508.2691, 480.2742, 476.2429, 448.2472, 354.2037, 105.0336
104	8-linolen-3,13-DDBA *	25.0	832.5358	832.5355	554.3107, 522.2846, 494.2897, 490.2581, 462.2634, 458.2313, 105.0333
105	8-linolen-DMDDBA *	25.0	802.5252	802.5237	524.2956, 492.2715, 464.2774, 460.2465, 432.2537, 105.0337
110	8-ole-DMBHA *	25.5	808.5358	808.5306	526.2795, 494.2547, 462.2274, 434.2291, 340.1912, 105.0336
114	8-pme-DBA *	25.9	824.5307	824.5305	570.3062, 538.2799, 510.2842, 506.2534, 478.2581, 352.1909, 105.0335
115	8-ole-13-DMDBA *	26.0	822.5515	822.5527	540.2964, 508.2698, 490.2586, 480.2745, 476.2435, 458.2323, 448.2480, 105.0336
116	8-pal-DMDBA *	26.0	796.5358	796.5371	540.2964, 508.2698, 490.2581, 480.2744, 476.2430, 458.2321, 448.2477, 105.0337
117	8-ole-3-DMDBA *	26.1	822.5515	822.5511	540.2965, 508.2700, 480.2479, 476.2441, 448.2477, 354.2071, 105.0336
119	8-pal-13-DMDBA *	26.1	796.5358	796.5368	540.2915, 508.2653, 480.2704, 476.2398, 448.2432, 354.2041, 105.0329
120	8-lino-DMDDBA *	26.2	804.5409	804.5387	524.3002, 492.2733, 464.2781, 460.2476, 432.2520, 105.0338
131	8-ole-DMDDBA *	27.4	806.5565	806.5512	524.2962, 492.2705, 464.2767, 460.2437, 432.2524, 105.0333
134	8-ole-3,13-DDBA *	27.7	836.5671	836.5619	554.3075, 522.2823, 494.2863, 490.2547, 462.2616, 458.2304, 105.0329
136	8-tcn-BMA *	28.2	926.6352	926.6308	572.2843, 540.2595, 522.2468, 512.2651, 508.2327, 490.2210, 476.2034, 354.1692, 105.0335
137	8-pal-DMDDBA *	28.3	780.5409	780.5384	524.3000, 492.2736, 464.2792, 460.2480, 432.2507, 105.0334
140	8-dcn-BMA *	28.9	912.6195	912.6194	572.2860, 540.2623, 522.2484, 512.2608, 508.2330, 490.2237, 480.2362, 354.1732, 105.0336

* Abbreviations: fmr (fumaric acid), bte (butenoic acid), hbte (hydroxybutenoic acid), scn (succinic acid), btn (butanoic acid), hbtn (hydroxybutanoic acid), dhbtn (dihydroxybutanoic acid), gtn (glutaconic acid), ogtr (oxoglutaric acid), gtr (glutaric acid), vlr (valeric acid), hvlr (hydroxyvaleric acid), act (aconitic acid), adp (adipic acid), hadp (hydroxyadipic acid), hxn (hexanoic acid), dohpnd (dioxoheptanedioic acid), otn (octanoic acid), hnne (hydroxynonenoic acid), azl (azelaic acid), nnn (nonanoic acid), sbc (sebacic acid), dhudn (dihydroxyundecanoic acid), dded (dodecenedioic acid), laur (lauric acid), myr (myristic acid), ptde (pentadecenoic acid), ptdn (pentadecanoic acid), pmde (palmitadienoic acid), pme (palmitoleic acid), pal (palmitic acid), hpdde (heptadecadienoic acid), hpde (heptadecenoic acid), hpdn (heptadecanoic acid), linolen (linolenic acid), hodt (hydroxyoctadecatrienoic acid), lino (linoleic acid), hodd (hydroxyoctadecadienoic acid), dhodd (dihydroxyloctadecadienoic acid), ole (oleic acid), hode (hydroxyoctadecenoic acid), dhode (dihydroxyoctadecenoic acid), thode (trihydroxyoctadecenoic acid), str (stearic acid), hstr (hydroxystearic acid), dhstr (dihydroxy stearic acid), thnde (trihydroxynonadecenoic acid), dhecte (dihydroxyeicosatrienoic acid), ecde (eicosadienoic acid), dhhctte (dihydroxyhenicosatetrenoic acid), dcn (docosanoic acid), tcn (tricosanoic acid), ttcn (tetrecosanoic acid), gvl (glucovanillic acid), gcf (glucocaffeic acid), gfr (glucoferilic acid).

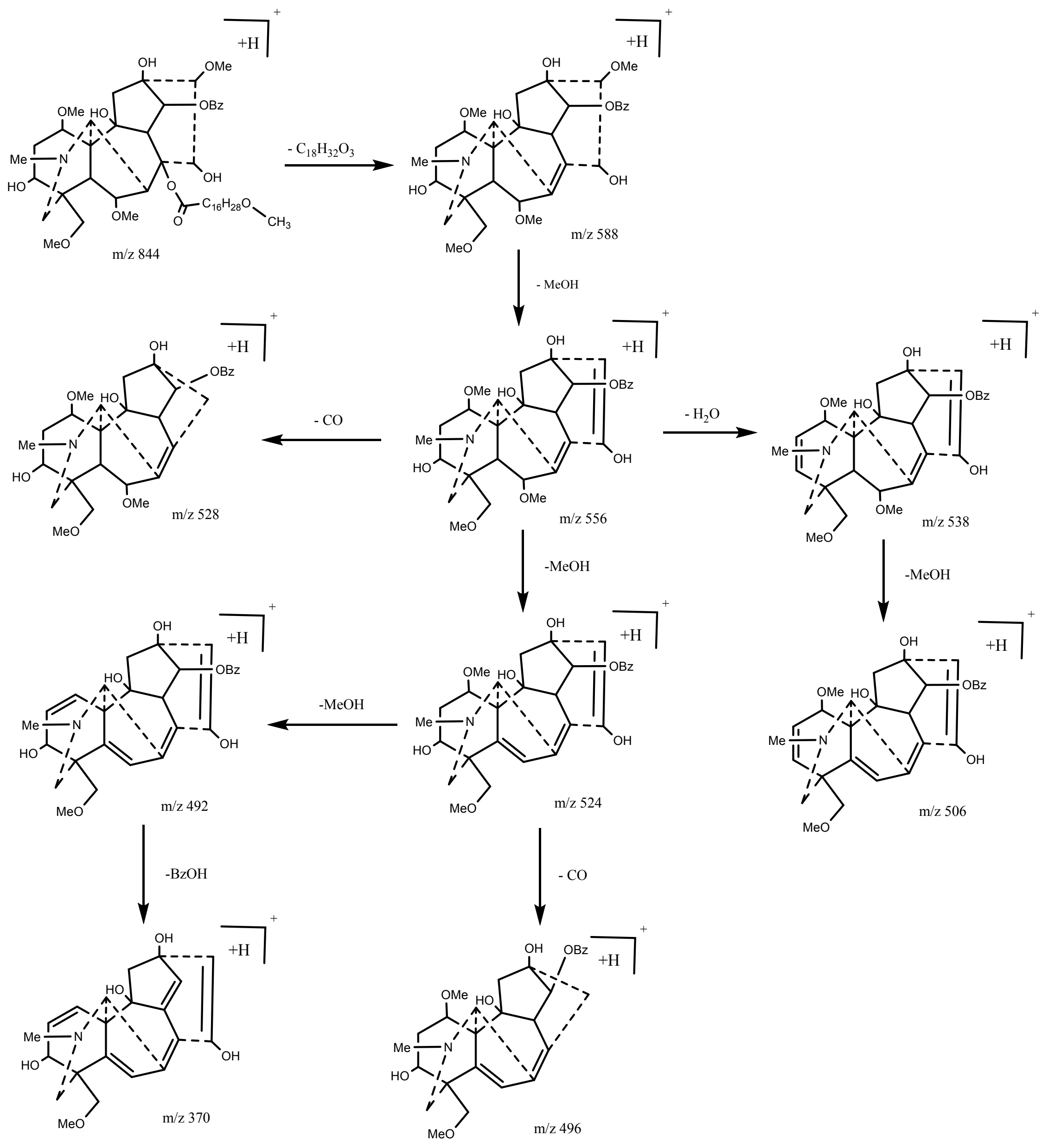

Scheme S1. Proposed fragmentation pathway of compound **62** in ESI-MS/MS.

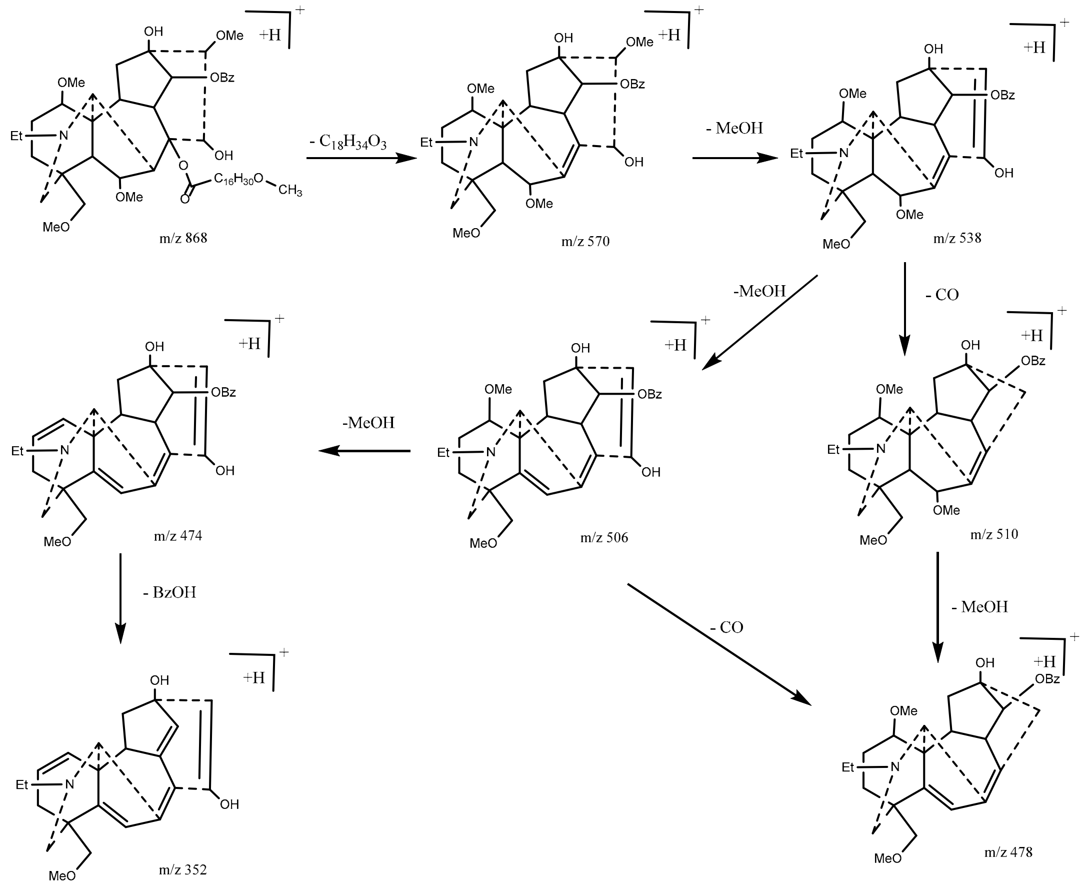

Scheme S2. Proposed fragmentation pathway of compound **90** in ESI-MS/MS.

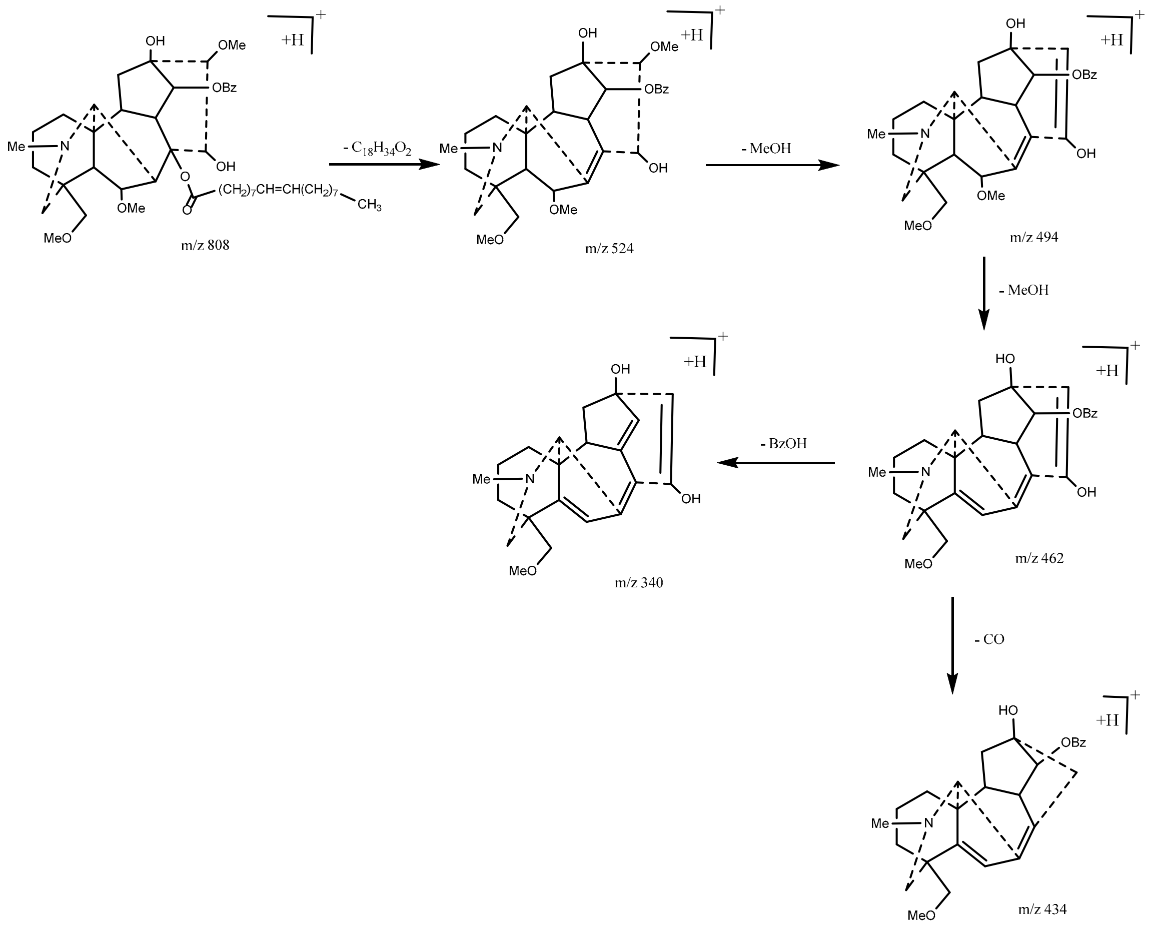

Scheme S3. Proposed fragmentation pathway of compound **110** in ESI-MS/MS.

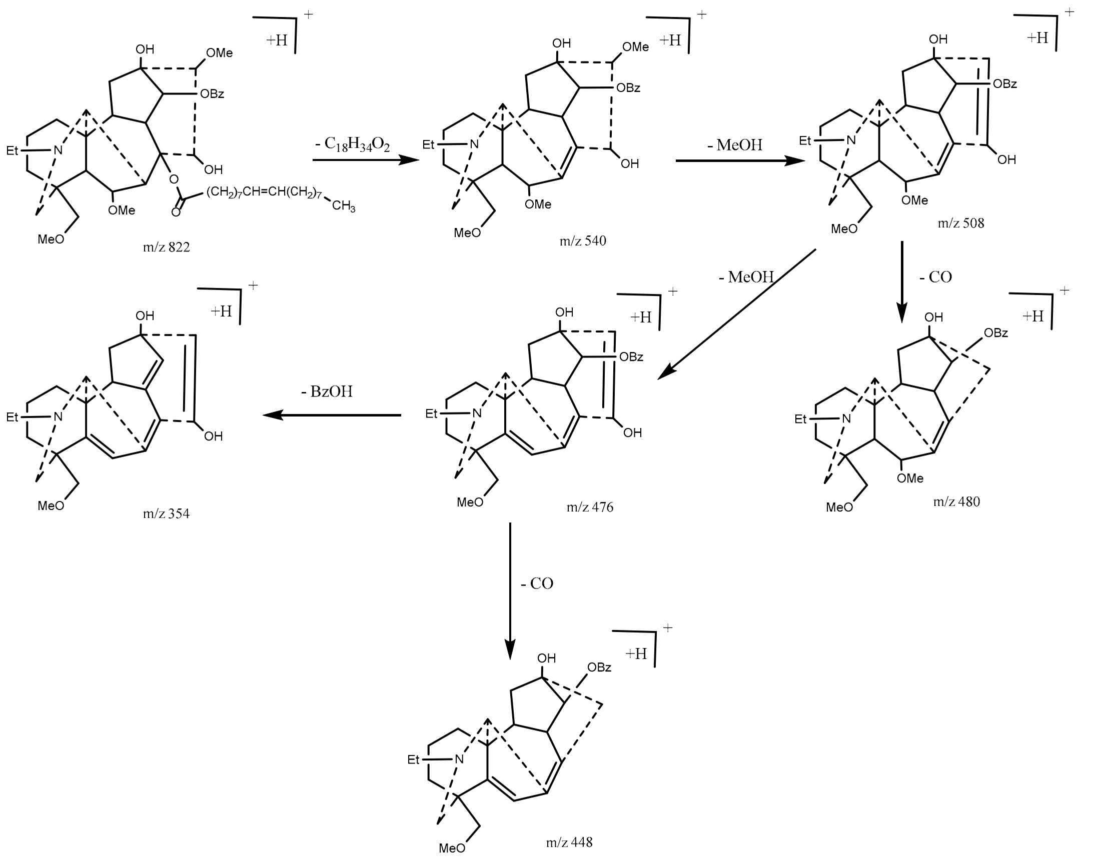

Scheme S4. Proposed fragmentation pathway of compound **117** in ESI-MS/MS.

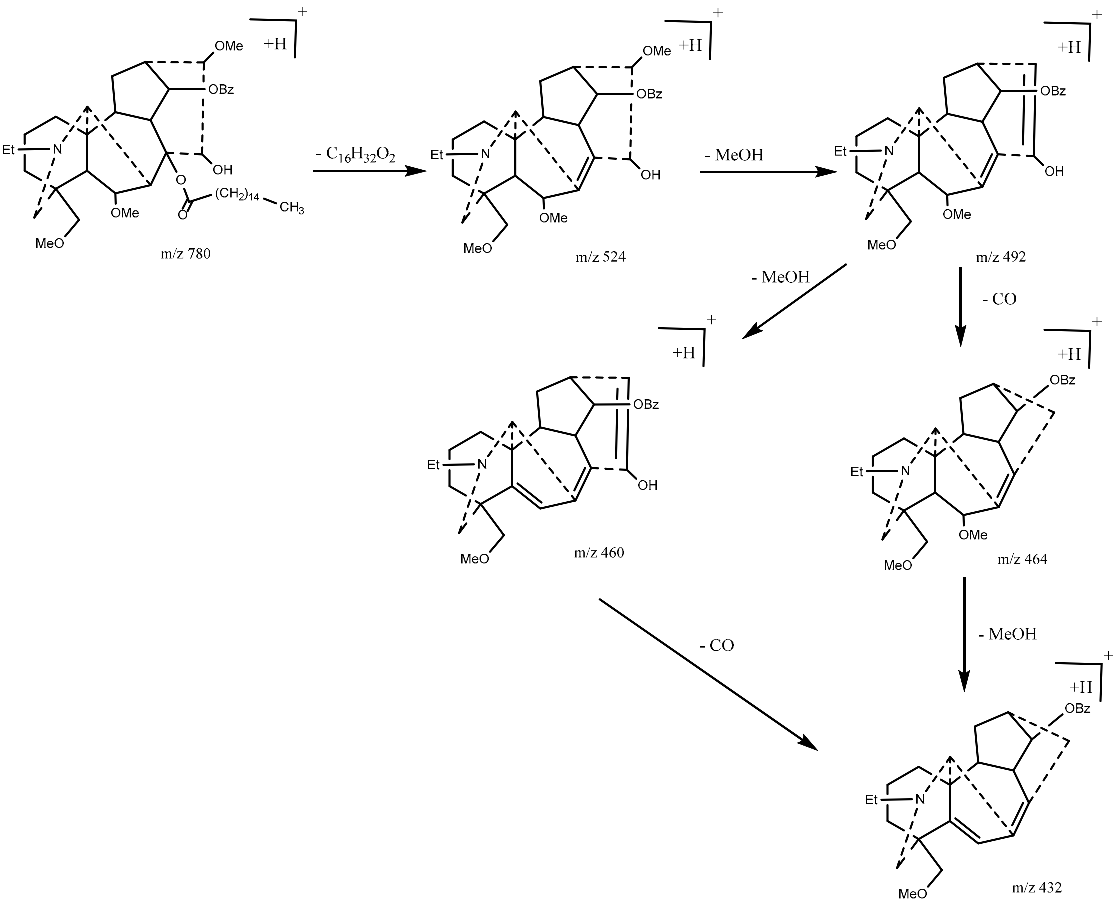

Scheme S5. Proposed fragmentation pathway of compound **137** in ESI-MS/MS.
